# Differential Diagnosis of Latent Tuberculosis Infection and Active Tuberculosis: A Key to a Successful Tuberculosis Control Strategy

**DOI:** 10.3389/fmicb.2021.745592

**Published:** 2021-10-22

**Authors:** Wenping Gong, Xueqiong Wu

**Affiliations:** Tuberculosis Prevention and Control Key Laboratory/Beijing Key Laboratory of New Techniques of Tuberculosis Diagnosis and Treatment, Senior Department of Tuberculosis, The 8th Medical Center of PLA General Hospital, Beijing, China

**Keywords:** tuberculosis, latent TB infection (LTBI), tuberculin skin test (TST), interferon-gamma release assays (IGRAs), biomarkers, differential diagnosis

## Abstract

As an ancient infectious disease, tuberculosis (TB) is still the leading cause of death from a single infectious agent worldwide. Latent TB infection (LTBI) has been recognized as the largest source of new TB cases and is one of the biggest obstacles to achieving the aim of the End TB Strategy. The latest data indicate that a considerable percentage of the population with LTBI and the lack of differential diagnosis between LTBI and active TB (aTB) may be potential reasons for the high TB morbidity and mortality in countries with high TB burdens. The tuberculin skin test (TST) has been used to diagnose TB for > 100 years, but it fails to distinguish patients with LTBI from those with aTB and people who have received Bacillus Calmette–Guérin vaccination. To overcome the limitations of TST, several new skin tests and interferon-gamma release assays have been developed, such as the Diaskintest, C-Tb skin test, EC-Test, and T-cell spot of the TB assay, QuantiFERON-TB Gold In-Tube, QuantiFERON-TB Gold-Plus, LIAISON QuantiFERON-TB Gold Plus test, and LIOFeron TB/LTBI. However, these methods cannot distinguish LTBI from aTB. To investigate the reasons why all these methods cannot distinguish LTBI from aTB, we have explained the concept and definition of LTBI and expounded on the immunological mechanism of LTBI in this review. In addition, we have outlined the research status, future directions, and challenges of LTBI differential diagnosis, including novel biomarkers derived from *Mycobacterium tuberculosis* and hosts, new models and algorithms, omics technologies, and microbiota.

## Introduction

Tuberculosis (TB) is one of the top 10 causes of death and is the leading cause of death from a single infectious agent worldwide (Gong et al., 2018; WHO, 2020). According to the Global Tuberculosis Report 2020 released by the World Health Organization (WHO), more than 10 million new TB cases and 1.4 million deaths were reported to the WHO in 2019 (WHO, 2020).

To reverse the long-term unfavorable situation of TB prevention and treatment, the WHO formulated an ambitious End TB Strategy in 2015. The aim of this strategy was to reduce the mortality and incidence rates of TB by 90% and 80%, respectively, in 2025 from those reported in 2015 and aim for further reduction by 95% and 90% in 2035, respectively (Stop TB Partnership, 2015). Currently, the global incidence of TB is declining at an average rate of about 2% per year, which is not fast enough to reach the target of a 17% annual decline from 2025 to 2035 set by the End TB Strategy (Stop TB Partnership, 2015; Chaw et al., 2020).

A reason for this gap may be that the source of infection has not been effectively controlled, resulting in a large percentage of the population being infected with latent TB infection (LTBI). A previous study indicated that 23% of the world’s population (equivalent to 1.7 billion people) was estimated to be latently infected with *Mycobacterium tuberculosis*, and cases from three WHO regions (Southeast Asia, Western-Pacific, and Africa), which had the highest LBTI burdens, accounted for approximately 80% of all LTBI cases (Houben and Dodd, 2016). Although countries with high TB burdens pay great importance to the prevention, diagnosis, and treatment of active TB (aTB), the same cannot be said in terms of their emphasis on LTBI.

It has been reported that 5–10% of patients with LTBI will progress to aTB during their lifetime (Hauck et al., 2009; Chee et al., 2018; WHO, 2018). However, the risk is higher in immunocompromised individuals, such as in people living with human immunodeficiency virus (HIV), people with diabetes, people with coronavirus disease, infants, and young children (aged < 5 years) (Chee et al., 2018; Gong and Wu, 2020; WHO, 2020; Aspatwar et al., 2021). In 2019, the number of new TB cases in countries with high TB burdens accounted for 86.9% of the global number of new TB cases (WHO, 2020). The latest data indicate that China has the heaviest burden of LTBI worldwide, with about 350 million people latently infected with *M. tuberculosis* (Cui et al., 2020). These data indicate that a significant percentage of the population with LTBI and lack of differential diagnosis of LTBI and aTB may be potential reasons for the high TB morbidity and mortality in countries with a high TB burden. Therefore, countries with high TB burdens should consider increasingly emphasizing LTBI-related research and taking action to accelerate progress toward global milestones and targets for reductions in the burden of TB set for 2025, 2030, and 2035 (Floyd et al., 2018). Accurately identifying and intervening in cases of TB from the population, especially cases of LTBI, are key to reducing morbidity and mortality. Reaching the milestones of the End TB Strategy is also urgent. Eliminating TB is not feasible if there is no isolation of patients with bacterium-positive TB and as long as a large number of people with LTBI exist (Godoy, 2021).

In this review, we first clarified the concept and definition of LTBI and then explained the immunological mechanism of LTBI. We also reviewed the current technologies and methods for LTBI differential diagnosis, such as the tuberculin skin test (TST) and interferon-gamma release assays (IGRAs), by comparing their advantages and disadvantages. Finally, we have outlined the current research status, future directions, and challenges for LTBI differential diagnosis in the future, including novel biomarkers derived from *M. tuberculosis* and its host, new models, algorithms, omics technologies, and microbiota.

## Concept and Immunological Mechanisms of Latent Tuberculosis Infection

### Evolutionary History of the Definition of Latent Tuberculosis Infection

With developments in science and technology, the understanding of the definition of LTBI has been deepening continuously over a long period. The evolution of the definition of LBTI can be divided into three stages—macropathology, bacteriology, and immunology. In the early 19th century, Louis (1825) and Laennec (1826) found tubercles upon autopsy of asymptomatic patients who had no clinical manifestations of TB before death, and they used the term “latent” TB to describe this condition for the first time. Behr et al. (2021) summarized the definition of LTBI in the 19th century as “Latent TB is a postmortem diagnosis referring to a host with tuberculous pathology in the absence of symptoms.” In the 20th century, the definition of LTBI began to shift from a pathological description to bacteriological identification. As early as 1956, when McCune et al. (1956) identified the effect of pyrazinamide on mice, they accidentally found that the disappearance of *M. tuberculosis* in the organs of mice did not mean that *M. tuberculosis* was completely eliminated. In contrast, *M. tuberculosis* can still be identified in approximately one-third of mice treated with pyrazinamide for 90 days. The bacteriological concept of LTBI was first proposed as follows: “the infection is present but is hidden beyond the limits of diagnostic reach” or “the presence of tubercle bacilli in the animal tissues can no longer be demonstrated by the most elaborate techniques of microscopy, culture, or animal inoculation” (McCune et al., 1956; Mc, 1959). However, this description is only a clinical and bacteriological definition but does not reflect the nature of LTBI. Behr et al. (2021) summarized the definition of LTBI in the 20th century as “Latent TB remains a postmortem diagnosis but now referring to the tubercle bacilli recovered from autopsy tissue without TB pathology.” In the 21st century, the definition of LTBI has changed from postmortem pathological diagnosis in the 19th century and bacterial diagnosis in the 20th century to host immunological diagnosis. In 1999, the American Thoracic Society (ATS) issued guidelines for the treatment of TB and specifically addressed “latent tuberculosis infection” (ATS, 2000). For the first time, TB immunoreactivity tests such as the TST were used to diagnose latent tuberculosis infection. Eight years later, these guidelines were updated, and the definition of LTBI was modified to include “a state of persistent immune response to stimulation by *M. tuberculosis* antigens with no evidence of clinically manifest active TB” (Lewinsohn et al., 2017). This definition of LTBI was recognized by the WHO in their Guidelines on the Management of Latent Tuberculosis Infection (WHO, 2015). On July 15, 2021, Behr et al. (2021) summarized the evolution of the definition of LTBI in the 21st century as follows: “Latent TB refers to a host who is TB immunoreactive in the absence of TB disease.”

As mentioned above, the understanding of LTBI has gone through three stages: anatomical diagnosis in the 19th century, bacteriological diagnosis in the 20th century, and immunological diagnosis in the 21st century. However, currently, there is no distinction between a “latent infection” and “immunological memory” in the absence of TB infection. A previous study has found that most TB-immunoreactive individuals retain immunological memory after clearing *M. tuberculosis* infection, indicating TB immunoreactivity can persist after curative treatment (Behr et al., 2018, 2019). It was estimated that 24.4% of individuals with *M. tuberculosis* infection could self-clear mycobacteria within 10 years and 73.1% over a lifetime (Emery et al., 2021). Therefore, the number of people harboring live *M. tuberculosis* may be substantially lower than previously reported (Behr et al., 2021). The population with positive results of TST and IGRAs includes not only individuals with LTBI but also individuals with persistent TB immunoreactivity after curative treatment. Furthermore, individuals with LTBI are at risk of progression to active TB. Therefore, they are a potential source of future transmission and may benefit from treatment. In contrast, individuals with immunological memory but no *M. tuberculosis* infection are not at risk of progression to active TB. Therefore, they are not a source of future transmission from the initial exposure and would not benefit from treatment. Thus, these concerns should be overcome in the future.

**FIGURE 1:**
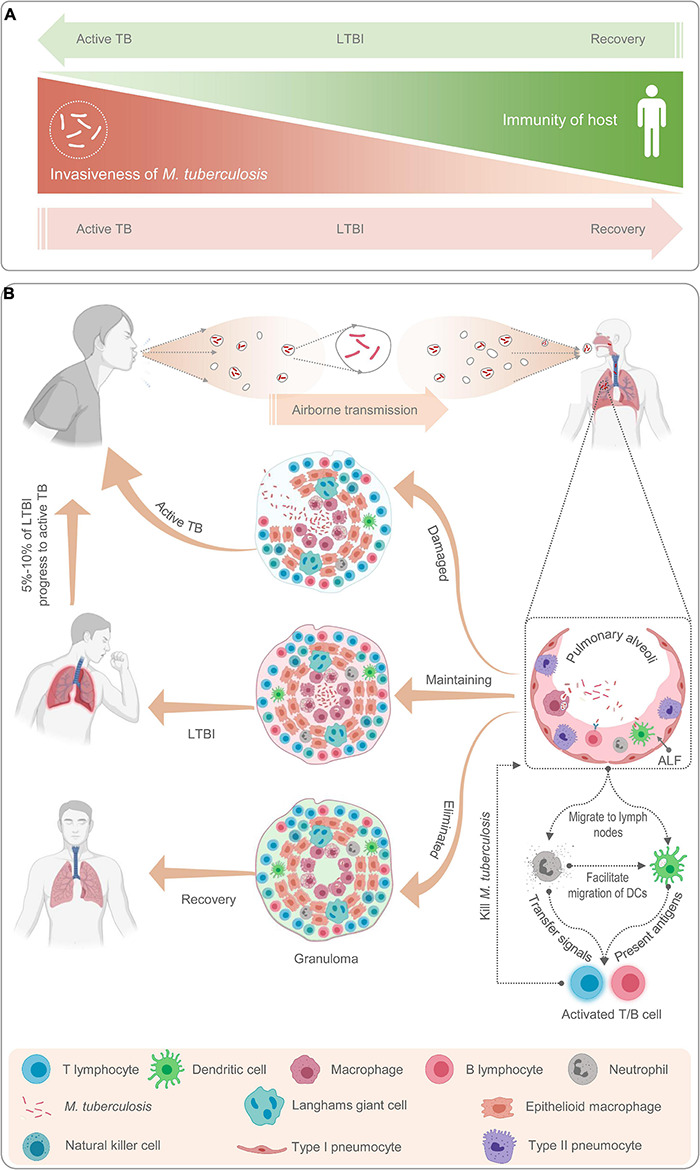
Schematic diagram of latent tuberculosis infection and its mechanism. Latent tuberculosis infection (LTBI) is a balance between immunity of host and invasiveness of *M. tuberculosis*; any tilt would upset the balance **(A)**. The *M. tuberculosis* excreted from patients with active TB is inhaled by healthy people through airborne transmission and recognized and phagocytized by antigen-presenting cells such as macrophages, neutrophils, natural killer cells, and B lymphocytes **(B)**. Then, neutrophils release cytokines to activate and recruit T lymphocytes to gather at the place where *M. tuberculosis* invades to form granulomas **(B)**. If the host immunity is strong, *M. tuberculosis* will be cleared by immune cells and the host recovers; If the host immunity is weak, *M. tuberculosis* will reproduce in the granulomatous tissue and breaks through the granulomatous restriction to cause active TB; If the immunity of host and invasiveness of *M. tuberculosis* is balanced, the host will be latently infected **(A,B)**.

In this review, we adopt the definition of LTBI from an article published by Chee et al. (2018) in 2018: “LTBI is a subclinical condition of *M. tuberculosis* infection, characterized by a complex and heterogeneous state resulting from the interaction between the immune response of the organism and the host.” In other words, we believe that LTBI is governed by a process of dynamic balance between host immunity and the invasiveness of *M. tuberculosis*. Once the balance is disrupted, *M. tuberculosis* infection can result in three outcomes (Figure 1A).

### Immunological Mechanisms of Latent Tuberculosis Infection

Host bactericidal immune responses play a central role in the progression from LTBI to aTB (Pan et al., 2017). To better understand the relationship between host immune responses and mycobacteria, we briefly reviewed the immunological mechanisms of LTBI (Figure 1B). As a paradigmatic intracellular pathogen, *M. tuberculosis* first invades the alveolar epithelial cells (pneumocytes I and II) after host inhalation of droplets containing bacteria *via* airborne transmission (Chai et al., 2020). The role of alveolar epithelial cells has been well documented in numerous studies since the 1990s (Kanai et al., 1979; Rodrigues et al., 2020). These cells play a crucial role in the pathogenesis of TB as replicative niches in the spread of infection *via* cellular death due to TB infection and play immunomodulatory roles through the expression of cytokines and chemokines (Scordo et al., 2016; Rodrigues et al., 2020). Macrophages are one of the first responders in host defense against *M. tuberculosis*. Inhaled *M. tuberculosis* is recognized and phagocytosed into phagosomes for clearance by resident alveolar macrophages mainly through their well-equipped pattern recognition receptors (PRRs), which can be blocked by some reactive proteins of *M. tuberculosis* (Westphalen et al., 2014). In addition, in response to *M. tuberculosis* infection, alveolar macrophages secrete a series of pro-inflammatory cytokines and chemokines, such as tumor necrosis factor-α (TNF-α), interleukin-1β (IL-1β), IL-6, IL-23, and granulocyte macrophage colony-stimulating factor (GM-CSF) (Chai et al., 2020). Lung-resident dendritic cells (DCs) are another group of sentinel cells that migrate to the surface of the trachea and alveoli to detect invading pathogens in time. Monocyte-derived DCs can recognize *M. tuberculosis* in collaboration with DC-specific intercellular adhesion molecule-3 grabbing non-integrin (DC-SIGN) and toll-like receptor (TLR) and kill *M. tuberculosis* by upregulating IL-1α, IL-1β, IL-10, and inducible nitric oxide synthase (iNOS) (Tailleux et al., 2003; Mayer-Barber et al., 2011; Chai et al., 2020). After phagocytosis *of M. tuberculosis*, DCs migrate to the surrounding lymph nodes and present the antigens of *M. tuberculosis* to prime CD4^+^ T lymphocytes (Chai et al., 2020).

Unlike macrophages and DCs, neutrophils do not recognize and phagocytose mycobacteria at the original infection sites. Neutrophils are important responders in host defense against *M. tuberculosis*, but they have been neglected until recently. Neutrophil recruitment occurs at an early stage of granuloma formation. In granulomas, the dying macrophages infected with *M. tuberculosis* release signals to recruit neutrophils to phagocytose them and *M. tuberculosis* (Yang et al., 2012). Previous studies revealed that neutrophils contribute to immune resistance to TB by producing antimicrobial peptides, facilitating the migration of DCs, and exerting protective effects on the oxidative killing of mycobacteria in TB granulomas (Martineau et al., 2007; Yang et al., 2012). Furthermore, accumulating evidence shows that B cells, natural killer (NK) cells, and mucosal-associated invariant T (MAIT) cells also play an important role in the immune response against *M. tuberculosis* by interacting with various immune cells, such as macrophages, T cells, and neutrophils (Achkar et al., 2015; Allen et al., 2015; Gong et al., 2021; Sakai et al., 2021).

However, the adaptive immune response is not triggered immediately; it has an interval of 2–3 weeks, which may be conducive to *M. tuberculosis* colonization (Jasenosky et al., 2015). Subsequently, T lymphocytes are activated and differentiate into interferon-γ-positive (IFN-γ^+^) Th1 cells (CD4^+^ T cells), cytotoxic T lymphocytes (CD8^+^ T cells), Th17 cells, Th2 cells, and regulatory T cells (Tregs) (Chai et al., 2020). These activated effector cells enter the blood circulation, migrate to the site of *M. tuberculosis* infection, and participate in local anti-TB immunity. At this stage, granulomas begin to form, and TST and IGRA results may be positive. The formation of tuberculous granulomas is a dynamic process between growth and decline. TB granulomas are the main battlefields for host immune cells and *M. tuberculosis*. The dying immune cells expose the hidden *M. tuberculosis* to the external environment to be phagocytosed and killed by the new immune cells. This process changes with the strength of the two belligerents, and the result directly affects the outcome of TB infection (Marino et al., 2015).

## Current Methods Used to Diagnosis Latent Tuberculosis Infection

It has been reported that approximately one-fourth of the global population is latently infected with *M. tuberculosis* and that 5–15% of them may progress to aTB within 2 years, while the remaining individuals with LTBI are at a constant risk of reactivation (Houben and Dodd, 2016; Pai and Behr, 2016). The differential diagnosis of individuals with LTBI can not only promote an understanding of the pathogenesis of TB but also reduce the risk of progression of LTBI to aTB through preventive treatment. However, there is no specific gold standard test for diagnosing LTBI thus far (Chee et al., 2018). Currently, the diagnosis of LTBI mainly depends on the host’s positive immune response to the antigens of *M. tuberculosis* and the clinical manifestations of the host. Currently, two methods are endorsed by the WHO as tests for TB infection—the century-old TST and IGRAs, which have been introduced since 2005 (Zellweger et al., 2020). The characteristics of the TST and IGRAs are listed in Table 1.

**TABLE 1:** The characteristics of the skin tests and IGRAs.

**Terms**	**Skin tests**				**IGRAs**				
	**Conventional TST**	**Diaskintest**	**C-Tb skin test**	**EC-Test**	**T-SPOT.TB**	**QFT-GIT**	**QFT-Plus**	**LIAISON QFT-Plus**	**LIOFeron TB/LTBI**
Manufacturer	Multiple manufacturers	Generium Pharmaceutical, Russia	Statens Serum Institut, Denmark	Zhifei Longcom, China	Oxford Immunotec, United Kingdom	QIAGEN, Hilden, Germany	Qiagen, MD, United States	DiaSorin S.p.A., Italy	LIONEX GmbH, Braunschweig, Germany
Time	> 100 years	2009	2010	2020	1990s	1980s	2015	2017	2019
Antigens	PPD	ESAT-6 and CFP10	ESAT-6 and CFP10	ESAT-6 and CFP10	ESAT-6 and CFP10	ESAT-6, CFP-10, and TB7.7 (p4)	ESAT-6 and CFP10	ESAT-6 and CFP10	ESAT-6, CFP-10, TB7.7, Ala-DH
Tubes	NA	NA	NA	NA	One tube	Nil tube, Antigen tube, and Mitogen tube	Nil tube, TB1 tube, TB2 tube, and Mitogen tube	Nil tube, TB1 tube, TB2 tube, and Mitogen tube	PC tube, TB-A tube, TB-B tube, and NC tube
Technological platform	NA	NA	NA	NA	ELISPOT	ELISA	ELISA	CLIA	ELISA
Sample	NA	NA	NA	NA	PBMCs	Whole blood	Whole blood	Whole blood	Whole blood
Sample transport and storage temperature	Refrigerate at 4°C	Refrigerate at 4°C	Refrigerate at 4°C	Refrigerate at 4°C	Indoor temperature, do not refrigerate or freeze	Indoor temperature, do not refrigerate or freeze	Indoor temperature, do not refrigerate or freeze	Indoor temperature, do not refrigerate or freeze	Indoor temperature, do not refrigerate or freeze
Outcome measure	Millimeters of induration	Millimeters of induration	Millimeters of induration	Millimeters of induration	Number of IFN-γ-producing T cells	Serum concentration of IFN-γ produced by CD4+ T cells	Serum concentration of IFN-γ produced by CD4+ and CD8+T cells	Serum concentration of IFN-γ produced by CD4+ and CD8+T cells	Serum concentration of IFN-γ produced by CD4+ and CD8+T cells
Positive internal control	No	No	No	No	PHA	Mitogen	Mitogen	Mitogen	Unknown
Need for return visit	Yes	Yes	Yes	Yes	No	No	No	No	No
Time required for results	48–72 h	48–72 h	48–72 h	48–72 h	16–20 h	16–24 h	16–24 h	Unknown	16–24 h
*in vivo*/*in vitro*	*in vivo*	*in vivo*	*in vivo*	*in vivo*	*in vitro*	*in vitro*	*in vitro*	*in vitro*	*in vitro*
Interpretation of result	Subjective (operator-based)	Subjective (operator-based)	Subjective (operator-based)	Subjective (operator-based)	Objective (instrument-based)	Objective (instrument-based)	Objective (instrument-based)	Objective (instrument-based)	Objective (instrument-based)
False positives with BCG vaccination	Yes	No	No	No	No	No	No	No	No
Cross-reactivity with NTMs	High	Low	Low	Low	Low, but can be influenced by infections of *M. kansasii*, *M. szulgai*, *M. marinum*, and M. gordonae*	Low, but can be influenced by infections of *M. kansasii*, *M. szulgai*, and *M. marinum* (Andersen et al., 2000)	Low, but can be influenced by infections of *M. kansasii*, *M. szulgai*, and *M. marinum* (Andersen et al., 2000)	Unknown	Low
False positives with immunosuppression and deficiency	High	Low	Low	Low	Low	Low	Low#	Unknown	Unknown
Specificity	62% (BCG vaccinated) and 95% (BCG unvaccinated) (Ruhwald et al., 2016)	98% (Starshinova et al., 2020)	99.3% (Aggerbeck et al., 2013)	98% (Steffen et al., 2020)	76.2% (Yang et al., 2021)	99%§	95% (Sotgiu et al., 2019)	Unknown	98% in aTB and 97% in LTBI (Della Bella et al., 2020)
Sensitivity	75% (Aggerbeck et al., 2018)†	86% (Starshinova et al., 2020)	73.9% (Hoff et al., 2016)	86% (Steffen et al., 2020)	83.5% (Yang et al., 2021)	89%§and 73% (Aggerbeck et al., 2018)†	91% (Sotgiu et al., 2019)	Unknown	90% in aTB and 94%in LTBI (Della Bella et al., 2020)
Accuracy	Unknown	95.1% in total population and 92.4% in HIV-positive patients (Starshinova et al., 2020)	Unknown	87%	88.5% (Yang et al., 2021)	Unknown	Unknown	Unknown	Unknown
Limitations	Relatively low specificity, lacks sensitivity in immunosuppressed individuals and requires two clinic visits	Requires two clinic visits	Requires two clinic visits	Requires two clinic visits, Safety need further observed, Data on LTBI high-risk populations and infants are lacking.	Results should be evaluated in conjunction with clinical and other tests, and a negative result does not rule out the possibility of infection with M. tuberculosis	Results must be used in conjunction with individual’s epidemiological history, current medical status, and other diagnostic evaluations	Results must be used in conjunction with risk assessment, radiography, and other medical and diagnostic evaluations	Need to define a borderline range based on clinical diagnostics criteria	Results must be used in conjunction with risk assessment, radiography, and other medical and diagnostic evaluations
Discrimination of LTBI from active TB (Hong et al., 2019; Della Bella et al., 2020; WHO, 2020)	No	No	No	No	No	No	No	No	No

*CLIA, chemiluminescence immunoassay; ELISPOT, enzyme-linked ImmunoSpot; IGRAs, interferon-gamma release assays; LTBI, latent tuberculosis infection; NA, not applicable; NC, negative control; NTMs, non-tuberculous mycobacteria; PBMCs, peripheral blood mononuclear cells; PC, positive control; PHA, phytohemagglutinin; PPD, protein-purified derivative; TST, tuberculin skin test.*

* These data were obtained from T-SPOT.TB official web (http://tspot.com.cn) and T-SPOT.TB ELISPOT Package Insert. PI-TB8-IVD-CN Rev. 05. September 2019. Accessed May 6, 2021.

*§These data were obtained from QuantiFERON official web (https://www.quantiferon.com/products/quantiferon-tb-gold/) and QuantiFERON-TB Gold (QFT) ELISA Package Insert. 1075115 Rev. 07. June 2018. Accessed May 6, 2021.*

*# Theoretically, the inclusion of peptides for stimulation of CD8^+^ T-cells can improve performance in immunocompromised conditions that affect CD4^+^ T-cell responses and improve discrimination of LTBI from active TB.*

*†Data are limited in children and HIV-infected persons.*

### Skin Tests

#### Conventional Tuberculin Skin Test

The TST is a diagnostic method performed through subcutaneous injection of old tuberculin (OT) or purified protein derivative (PPD) as an antigen (Mosavari et al., 2021). It has been applied in the screening, diagnosis, and epidemiological studies of primary infection with *M. tuberculosis* for more than 100 years and is the main diagnostic test for LTBI. The TST is widely used to screen and detect TB owing to its advantages of being affordable, being simple to perform, and the requirement of minimal laboratory equipment. However, its disadvantages cannot be neglected, such as its requirement for a second visit after 48–72 h, inability to distinguish LTBI from aTB, false positives with Bacillus Calmette–Guérin (BCG) vaccination, cross-reactivity with non-tuberculous mycobacteria (NTM), and false negatives in immunosuppression and deficiency (Pourakbari et al., 2019).

#### Novel Emerging Skin Tests

In addition to the conventional TST, three novel strategies for the diagnosis of TB have been developed in recent years—Diaskintest (Generium Pharmaceutical, Moscow, Russia), C-Tb skin test (Statens Serum Institut, Copenhagen, Denmark), and EC-Test (Zhifei Longcom Biologic Pharmacy Co., Anhui, China) (Steffen et al., 2020; WHO, 2020). The Diaskintest is a new effective way to identify the initial presentation of TB and developed based on a fusion protein of the early secreted antigenic target 6 (ESAT-6) and culture filtrate protein 10 (CFP-10) antigens present in virulent strains of *M. tuberculosis*, which are absent in the BCG strain and in most NTMs. Recently, a meta-analysis based on 61 articles and 3,777,083 patients was conducted to evaluate the sensitivity and accuracy of the Diaskintest, and the results indicated that the overall diagnostic specificity, sensitivity, and accuracy of the Diaskintest were 98.0, 86.0, and 95.1%, respectively (Starshinova et al., 2020). However, the C-terminal of the fusion protein used in the Diaskintest contains 11 amino acids (including six histidine (HIS) labels) on the vector sequence other than the target gene, which does not conform to the “Technical Guiding Principles for Quality Control of Recombined DNA Products for Human Use” issued by the China Food and Drug Administration. The novel C-Tb skin test is based on a mixture of antigens ESAT-6 and CFP10. It combines the advantages of the field friendliness of TST and the high specificity of IGRAs. Two phase III clinical trials have investigated the safety and efficacy of the C-Tb skin test, QuantiFERON-TB Gold In-Tube (QFT-GIT), and TST, suggesting that C-Tb and QFT-GIT results were concordant in 94% of participants and that the safety profile of the C-Tb skin test was similar to that of the TST and QFT-GIT in HIV-negative adults and children as well as HIV-positive individuals with active or latent *M. tuberculosis* infection (Ruhwald et al., 2017; Aggerbeck et al., 2018). The EC-Test is the latest skin test for the diagnosis of aTB and LTBI. Similar to the Diaskintest and C-Tb skin test, the EC-Test is also developed based on ESAT-6 and CFP10 antigens of *M. tuberculosis*. Currently, clinical phase I, II, and III trials of the EC-Test have been completed. A phase I clinical trial and a phase IIa clinical trial indicated that the median maximum induration diameter of the EC-Test was similar to that of the TST and that a single dose of the EC-Test (1, 5, 10, or 20 μg/ml) was well tolerated and safe, with its diagnostic accuracy superior to that of the TST (Li et al., 2016a, b). A phase III clinical trial conducted in 1,559 participants found that the EC-Test and IGRAs have fairly high specificity and consistency (88.77%) (Chinese Antituberculosis Association et al., 2020).

Taken together, these three new tests are designed to detect immune responses stimulated with recombinant ESAT-6 and CFP-10 antigens to help address the problem of false-positive TST results that occur in patients who have received BCG vaccination (Ruhwald et al., 2016). Their specificity may be higher than that of the TST and may be more accurate, acceptable, and cheaper alternatives to IGRAs (Ruhwald et al., 2017). These skin tests have the advantages of the TST and IGRAs, which are characterized by simple operation, low cost, and easy reading of results, making it possible to apply them in countries with a high burden of TB and in poverty-stricken areas, especially in countries implementing universal BCG vaccination. Furthermore, increasingly emerging studies have demonstrated that these new skin tests show similar test positivity rates, safety profiles, and specificities to those of IGRAs and provide comparable results in terms of diagnosing patients with TB living with HIV and children infected with *M. tuberculosis* (Table 1; Litvinov et al., 2009; Aggerbeck et al., 2013; Hoff et al., 2016; Ruhwald et al., 2017; Aggerbeck et al., 2018; Steffen et al., 2020).

Nevertheless, the potential drawbacks of these three new skin tests are worth noting. First, these skin tests have insufficient diagnostic foundations for the differential diagnosis of LTBI. Second, vaccines based on recombinant ESAT6-CFP10 protein for prevention of LTBI may affect the diagnostic value of these skin tests in the future. Finally, these three novel skin tests should be validated in larger clinical trials in children, elderly individuals, and special populations such as in HIV-infected persons, immunosuppressant drug users, and people with malnutrition.

### Interferon-Gamma Release Assays

To overcome the deficiencies of the TST, IGRAs have been developed as an alternative immunodiagnostic approach. Thus far, there are five commercialized IGRA kits, including the T-cell spot of the TB assay (T-SPOT.TB, Oxford Immunotec, Abingdon, UK), QuantiFERON-TB Gold In-Tube (QFT-GIT, Qiagen GmbH, Hilden, Germany), QuantiFERON-TB Gold-Plus (QFT-Plus, Qiagen, MD, United States), LIAISON QuantiFERON-TB Gold Plus (LIAISON QFT-Plus, DiaSorin S.p.A., Italy), and LIOFeron TB/LTBI (LIONEX GmbH, Braunschweig, Germany) (De Maertelaere et al., 2020; Della Bella et al., 2020; Altawallbeh et al., 2021; Hamada et al., 2021). In addition, there are five products in development—T-Track (R) TB (Lophius Biosciences GmbH), VIDAS TB-IGRA (bioMérieux), Access QuantiFERON-TB (Boditech Med Inc.), ichroma^TM^ IGRATB (Boditech Med Inc.), and IP-10 IGRA elisa/lateral flow (rBioPharm) (WHO, 2020).

#### T-cell Spot of the Tuberculosis Assay, QuantiFERON- Tuberculosis Gold In-Tube Test, and QuantiFERON- Tuberculosis Gold-Plus

T-cell spot of the TB assay is an enzyme-linked immunospot (ELISpot) assay developed by Prof. Lalvani in collaboration with his colleagues in the late 1990s (Lalvani et al., 2001). T-SPOT.TB detects the immune responses of peripheral blood-derived IFN-γ-secreting T cells stimulated with peptides from ESAT-6 and CFP10 antigens. The QFT-GIT assay is based on stimulating IFN-γ release from CD4^+^ T cells in a single TB antigen tube with three antigens of *M. tuberculosis*, including ESAT-6, CFP-10, and TB7.7. Antigens ESAT-6 and CFP-10 are encoded within the *M. tuberculosis* region of difference 1 (RD1) locus, which eliminates interference from BCG and NTMs (Saracino et al., 2009). The QFT-Plus assay is designed to stimulate CD4^+^ T cells using ESAT-6 and CFP-10 antigens in tube 1 (TB1) and elicit IFN-γ release from both CD4^+^ and CD8^+^ T cells using a cocktail of peptides derived from ESAT-6 and CFP-10 antigens in TB2 (Rozot et al., 2013). Compared with the QFT-GIT assay based on CD4^+^ T-cell immune responses, the biggest improvement in the QFT-Plus assay is the addition of CD8^+^ T-cell responses. It has been reported that the CD8^+^ T-cell response in patients with aTB is higher than that in patients with LTBI (Theel et al., 2018). A systematic review and meta-analysis demonstrated that QFT-Plus is more sensitive than QFT-GIT for detecting *M. tuberculosis* infection (Sotgiu et al., 2019), which is consistent with the results of another meta-analysis (Pourakbari et al., 2019). These data suggest that QFT-Plus is more sensitive than QFT-GIT in detecting *M. tuberculosis* infection, which may be due to CD8^+^ T-cell immune responses in the TB2 tube. Therefore, QFT-Plus was approved by the United States Food and Drug Administration in 2017 to replace QFT-GIT (Theel et al., 2018). In 2019, Venkatappa et al. (2019) evaluated the agreement between QFT-Plus, QFT-GIT, T-SPOT.TB, and the TST in 506 participants at a high risk of LTBI and/or progression to TB disease. Their results showed that there was 94% agreement between QFT-Plus and QFT-GIT, 77% agreement between QFT-Plus or QFT-GIT and the TST, 92% agreement between QFT-Plus and SPOT.TB, and 91% agreement between QFT-GIT and T-SPOT.TB. These results indicated a high degree of agreement between QFT-GIT and QFT-Plus in a direct comparison and that all tests were in agreement with the TST and T-SPOT.TB.

#### LIAISON QuantiFERON- Tuberculosis Gold-Plus

The LIAISON QFT-Plus test, developed by DiaSorin in collaboration with Qiagen, is a whole blood-based QuantiFERON technology that offers efficient and high-throughput detection with QFT-Plus. The LIAISON QFT-Plus test is a fourth-generation technology for detecting LTBI. It is an improved version of the QFT-Plus test, in which the enzyme-linked immunosorbent assay (ELISA) in QFT-Plus has been replaced by a chemiluminescent immunoassay (CLIA) (Altawallbeh et al., 2021). A recent study evaluated the diagnostic performance of LIAISON QFT-Plus by comparing it with that of QFT-Plus test in 329 participants, showing 92.8, 97.9, and 97.8% agreement with the QFT-Plus test among patients with aTB, low-risk cohort, and mixed risk cohort participants, respectively (Altawallbeh et al., 2021). Furthermore, LIAISON QFT-Plus test can save time and labor and be a potentially useful addition to streamline LTBI screening (Kadkhoda et al., 2020). Moreover, these findings suggest that the automated LIAISON QFT-Plus test has a diagnostic performance comparable to that of the QFT-Plus test and can be applied for LTBI diagnosis. However, given that it is a newly emerging technology, the LIAISON QFT-Plus test requires more studies with large sample sizes to prove its sensitivity and specificity in the future.

#### LIOFeron Tuberculosis/Latent Tuberculosis Infection Assay

The LIOFeron TB/LTBI assay, developed by Lionex GmbH (Braunschweig, Germany) in 2019, is a novel IGRA test for diagnosing LTBI and TB (LIONEX, 2021). The LIOFeron TB/LTBI assay contains two components—human blood stimulation tubes (component 01) and human IFN-γ ELISA (component 02). Component 01 contains a positive control tube, a negative control tube, and TB antigen tubes. Compared with other IGRAs, the LIOFeron TB/LTBI assay contains not only ESAT-6, CFP-10, and TB7.7 but also a new antigen of *M. tuberculosis*, alanine dehydrogenase (Ala-DH). Previous studies have reported that Ala-DH is absent in BCG, induces major histocompatibility complex (MHC) class I-restricted T CD8^+^ lymphocytes to produce IFN-γ, and participates in the adaptation of *M. tuberculosis* to the anaerobic dormant stage in LTBI (Jungblut et al., 1999; Dong et al., 2004; Agren et al., 2008). Recently, the sensitivity and specificity of the novel test were evaluated, and a comparison between its accuracy and that of the QFT-PLUS assay was performed in patients with aTB and LBTI (Della Bella et al., 2020). The results demonstrated that the sensitivity and specificity of the LIOFeron TB/LTBI assay was 90% and 98% for diagnosing aTB and 94% and 97% for diagnosing LTBI and that the LIOFeron TB/LTBI assay showed higher sensitivity than the QFT-Plus test for LTBI detection (Della Bella et al., 2020). However, like other IGRAs, the LIOFeron TB/LTBI assay cannot differentiate between LTBI and aTB.

## Research Status, Future Directions, and Challenges for Latent Tuberculosis Infection Differential Diagnosis

Current methods such as the Diaskintest, C-Tb skin test, EC-Test, and T-SPOT.TB, QFT-GIT, QFT-Plus, and LIAISON QFT-Plus have greatly improved the accuracy, sensitivity, and specificity of *M. tuberculosis* infection detection, but they are still unable to distinguish LTBI from aTB. Since LTBI is a complicated heterogeneous state reflecting the interaction between *M. tuberculosis* and the host’s immune response, the development of a better diagnostic technology that can differentiate LTBI from aTB requires a deep understanding of both the mycobacteria and its host (Chee et al., 2018). Herein, we will start from these two aspects to outline a possible blueprint for the technology exploration of LTBI differential diagnosis in hopes of pointing out directions and laying the foundation for future research on the topic.

### Novel Biomarkers Derived From *Mycobacterium tuberculosis*

#### Antigens

*Mycobacterium tuberculosis* proteins encoded by RDs are feasible candidate biomarkers for differentiating *M. tuberculosis* infection from BCG vaccination (Al-Khodari et al., 2011). As mentioned above, the Diaskintest, C-Tb skin test, EC-Test, and T-SPOT.TB, QFT-GIT, QFT-Plus, and LIAISON QFT-Plus were designed based on RD1-encoded antigens, such as ESAT-6 and CFP-10. After reading the literature, we found 129 RD-associated antigens within the 16 RDs of *M. tuberculosis* (Ren et al., 2018). Additionally, although four antigens (Rv1737c, Rv1736c, Rv2031c, and Rv2626c) were excluded from the RD regions, previous studies have shown that these antigens show significant differences between the BCG strain and *M. tuberculosis* (Geluk et al., 2007; Honaker et al., 2008; Ji et al., 2015; Pena et al., 2015). Therefore, all 133 RD-associated antigens should be taken into consideration for developing new methods for the differential diagnosis of LTBI, with detailed information found in Supplementary Table 1. As mentioned above, none of these seven novel methods can distinguish LTBI from aTB, suggesting that the differential diagnosis method of LTBI should contain not only RD-associated antigens but also latency-associated *M. tuberculosis* antigens.

Herein, we suggest that IGRAs should be further improved by the application of multiple antigens derived from RD-associated and latency-associated antigens of *M. tuberculosis*. Numerous studies have aimed to identify latency-associated antigens from *M. tuberculosis* as potential candidates for inclusion in immunodiagnostic tests for LTBI. A total of 124 latency-associated antigens were found in six categories—54 dormancy survival regulon antigens (DosRs), seven nutrition starvation (NS)-associated antigens, 20 reactivation antigens (RAs), five resuscitation-promoting factor (RPF) antigens, eight toxin–antitoxin system-associated antigens (TAS), and 30 other antigens associated with LTBI (Supplementary Table 2; Gordon et al., 2001; Chen et al., 2009; Ji et al., 2015; Meier et al., 2018).

**FIGURE 2:**
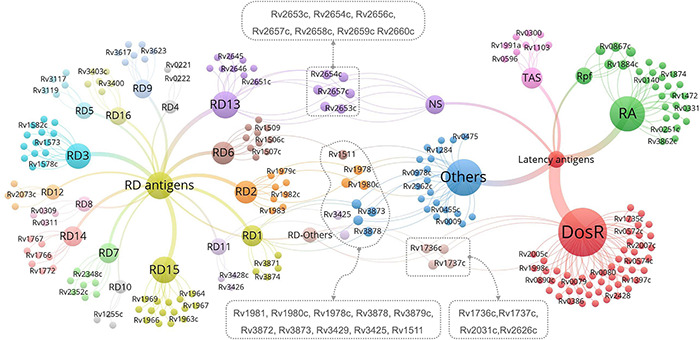
The most promising LTBI-RD-related antigens for differential diagnosis of latent tuberculosis infection (LTBI) in the future. LTBI-related antigens are divided into six subtypes, including DosR, TAS, RA, NS, Rpf, and Others. RD-related antigens are divided into 16 subtypes, including RD1-RD15 and Others. Each subtype contains several antigens, each antigen is represented by a bubble, and different subtypes are represented by different colors. The size of the bubble represents the number of antigens in the subtype, and the line thickness represent the strength of the connection. The LTBI-RD-related antigens were identified by using a visualization and exploration software Graph (version 0.9.2, https://gephi.org/) and highlighted with dotted box. Each dot presents an antigen, limited to the space, some antigens’ names were not shown in this figure, the detailed information can be found in Supplementary Tables 1, 2.

Antigens belonging to both latency and RDs are the most novel and promising targets for LTBI differential diagnosis. Thus, we identified 21 LTBI-RD-related antigens from 133 RD-associated antigens and 124 latency-associated antigens of *M. tuberculosis*, including Rv1736c, Rv1737c, Rv2031c, Rv2626c, Rv2653c, Rv2654c, Rv2656c, Rv2657c, Rv2658c, Rv2659c, Rv2660c, Rv1511, Rv1978, Rv1980c, Rv1981c, Rv3872, Rv3873, Rv3878, Rv3879c, Rv3425, and Rv3429 (Figure 2). Detailed information on these 21 LTBI-RD-related antigens is summarized in Table 2. In brief, (1) There are four antigens derived from DosRs and RD-others, including Rv1736c, Rv1737c, Rv2031c, and Rv2626c. Honaker et al. (2008) reported that Rv1736c and Rv1737c were induced by *M. tuberculosis* rather than 13 BCG strains or *M. bovis*, suggesting that both antigens can be used to differentially diagnose latent infection even in BCG-vaccinated individuals. Rv1737c induced higher frequencies of IFN-γ^+^ CD8^+^ T cells and IFN-γ^+^ TNF-α^+^ CD4^+^ T cells with a CD45RO^+^ CD27^+^ phenotype in long-term LTBI than pulmonary TB (PTB) (Arroyo et al., 2016), and the levels of the five cytokines [IL-10, transforming growth factor (TGF)-α, TNF-α, IL-12 (p40), and epidermal growth factor (EGF)] stimulated by Rv1737c were significantly higher in patients with TB than in household contacts (Chegou et al., 2012). For the Rv2031c antigen, it has been indicated that it could induce significantly higher IFN-γ production in the TST^–^/RD1^+^ and TST^+^/RD1^+^ groups than in the aTB group (Araujo et al., 2015). Furthermore, in patients with long-term LTBIs, higher percentages of IFN-γ^+^ TNF-α^+^ CD8^+^ T cells were found in response to Rv2031c protein in comparison with PPD^–^ controls, with the most frequent memory phenotype of these cells being effector memory (CCR7^–^ CD45RA^–^) T cells, followed by effector (CCR7^–^ CD45RA^+^) T cells (Commandeur et al., 2011), which is consistent with an essential role of CD8^+^ T cells in defending the host from *M. tuberculosis* infection (Bruns et al., 2009). The Rv2626c antigen induced significantly higher levels of IFN-γ in QFT^+^ individuals or healthy household contacts than in QFT^–^ individuals or patients with TB and had the ability to differentiate individuals with LTBI from healthy controls and patients with TB, with 78.95% sensitivity and 83.02% specificity (Prabhavathi et al., 2015; Amiano et al., 2020), which is consistent with the sensitivity (77.1%) and specificity (85.1%) of Rv2626c reported in another study (Ashraf et al., 2014).

**TABLE 2:** Summary of the most important LTBI-RD related antigens for LTBI differential diagnosis.

**Antigens^a^**	**Types^a^**		**Product ^b^**	**Function^b^**	**Functional category^b^**	**LTBI differential diagnosis**	**References**
	**LTBI**	**RD**					
Rv1736c	DosR	Others	Probable nitrate reductase NarX	Involved in nitrate reduction, and in the persistence in the host	Intermediary metabolism and respiration	Induced by *M. tuberculosis* rather than 13 BCG strains or *M. bovis*	Honaker et al., 2008
Rv1737c	DosR	Others	Possible nitrate/nitrite transporter NarK2	Involved in excretion of nitrite, produced by the dissimilatory reduction of nitrate, across the membrane. Responsible for the translocation of the substrate across the membrane.	Cell wall and cell processes	(1) Higher TNF-α^+^ CD4^+^ T cells and IFN-γ^+^ TNF-α^+^ CD4^+^ T cells in LTBI vs. PTB. (2) Higher IFN-γ^+^ TNF-α^+^ CD8^+^ T cells in LTBI vs. HC	Chegou et al., 2012; Arroyo et al., 2016
Rv2031c	DosR	Others	Heat shock protein HspX	Has a proposed role in maintenance of long-term viability during latent, asymptomatic infections, and a proposed role in replication during initial infection.	Virulence, detoxification, adaptation	(1) Higher IFN-γ^+^ TNF-α^+^ CD8^+^ T cells in LTBI vs. HCs (2) Higher concentrations of IFN-γ in LTBI vs. aTB vs. HC (3) Lower IFN-γ, IL-10, TNF-α in LTBI vs. aTB and HCs	Commandeur et al., 2011; Araujo et al., 2015; Belay et al., 2015
Rv2626c	DosR	Others	Hypoxic response protein 1 Hrp1	Unknown	Conserved hypotheticals	(1) 91% of CC QFT^–^ subjects secreted low levels of IFN-γ, but 43% of HCWs QFT^–^ people produced elevated IFN-γ, 69% of CC QFT^+^ subjects didn’t produce IFN-γ to Rv2626c (2) Higher IFN-γ producing T cells in CC vs. aTB and HC (3) IFN-γ response to Rv2626c has shown positivity of 88.57% in CC and 7.5% in PTB (4) Sensitivity and specificity of Rv2626c in aTB was of 77.1% and 85.1%	Ashraf et al., 2014; Prabhavathi et al., 2015; Amiano et al., 2020
Rv2653c	NS	RD-13	Possible PhiRv2 prophage protein	Unknown	Insertion seqs and phages	Less reactive DTH skin responses in *M. tuberculosis*-sensitized guinea pigs vs. PPD, but elicited no response in BCG-vaccinated guinea pigs.	Kasempimolporn et al., 2019
Rv2654c	NS	RD-13	Possible PhiRv2 prophage protein	Unknown	Insertion seqs and phages	(1) Higher IFN-γ levels TB vs. HC, had a high overall agreement (98.0%) with T-SPOT.TB, the combination of Rv2645 and CFP10-ESAT6 increased sensitivity and specificity of 96.0% and 98.2%, respectively. (2) Peptide Rv2654c_51–65_ boosted the quantitative performance of the QFT-GIT assay from 1.83 IU/ml to 2.83 IU/ml	Luo et al., 2015; Horvati et al., 2016
Rv2656c	NS	RD-13	Possible PhiRv2 prophage protein	Unknown	Insertion seqs and phages	Unknown	-
Rv2657c	NS	RD-13	Probable PhiRv2 prophage integrase	Unknown	Insertion seqs and phages	Rv2657c react with the sera from LTBI Guinea pigs but not healthy Guinea pigs, SFCs of HCWs was significantly higher in HCWs vs. aTB	Yang, 2018
Rv2658c	NS	RD-13	Possible prophage protein	Unknown	Insertion seqs and phages	Unknown	-
Rv2659c	NS	RD-13	Probable PhiRv2 prophage integrase	Sequence integration. Integrase is necessary for integration of a phage into the host genome by site-specific recombination	Insertion seqs and phages	Higher IFN-γ producing T cells in LTBI vs. aTB and HC	Bai, 2015
Rv2660c	NS	RD-13	Hypothetical protein	Unknown	Conserved hypotheticals	(1) Higher number of IFN-γ producing cells and the levels of cytokines (IFN-γ, IL-2, IL-10, and MIP-1621299821α 621299821) in LTBI vs. TB, and higher ratio of IFN-γ+ CD4+ T cells in LTBI vs. TB or HCs (2) A component of vaccine H56:IC31 and will affect the diagnosis of Rv2660c once the vaccine is available	He et al., 2015; Luabeya et al., 2015
Rv1511	Others	RD-6	GDP-D-mannose dehydratase GmdA	Unknown, probably involved in nucleotide-sugar metabolism	Intermediary metabolism and respiration	Unknown	-
Rv1978	Others	RD-2	Conserved protein	Unknown	Conserved hypotheticals	ELISPOT of Rv1978 achieved sensitivities of 59% in aTB and specificities of 94% in BCG-vaccinated HC.	Chen et al., 2009
Rv1980c	Others	RD-2	Immunogenic protein Mpt64	Unknown	Cell wall and cell processes	Sensitivity and specificity were 0.92 and 0.95, respectively, sensitivity of the MPT64 test was significantly higher in TB infected children than in adults.	Cao et al., 2021
Rv1981c	Others	RD-2	Ribonucleoside-diphosphate reductase (beta chain) NrdF1	Involved in the DNA replication pathway.	Information pathways	ELISPOT of Rv1981c achieved sensitivities of 60% in aTB and specificities of 90% in BCG-vaccinated HC.	Chen et al., 2009
Rv3872	Others	RD-1	PE family-related protein PE35	Unknown	Pe/ppe	(1) Sensitivity and specificity of PE35 for detecting LTBI in children were 76% and 80%. (2) Elicited stronger immunoreactivity and could discriminate TB from HC vaccinated with BCG (better than Rv3878).	Mukherjee et al., 2007; Mahmoudi et al., 2017
Rv3873	Others	RD-1	PPE family protein PPE68	Unknown	Pe/ppe	Sensitivity and specificity of PE68 for detecting LTBI in children were 73% and 75%.	Mahmoudi et al., 2017
Rv3878	Others	RD-1	ESX-1 secretion-associated protein EspJ	Unknown	Cell wall and cell processes	Elicited stronger immunoreactivity and could discriminate TB from HC vaccinated with BCG.	Mukherjee et al., 2007
Rv3879c	Others	RD-1	ESX-1 secretion-associated protein EspK	Unknown	Cell wall and cell processes	The immunodominance of Rv3879c is higher than that of Rv3878 and Rv3873 in aTB and LTBI subjects.	Hinks et al., 2009
Rv3425	Others	RD-11	PPE family protein PPE57	Unknown	Pe/ppe	(1) Rv0310c-E coupled with Rv3425 (sensitivity: 87.30%, specificity: 73.68%) had the strongest performance for diagnostics of aTB. (2) Rv3425 has the promising potential to distinguish aTB from HC vaccinated with BCG.	Zhang et al., 2007; Wang et al., 2013; Luo et al., 2017
Rv3429	Others	RD-11	PPE family protein PPE59	Unknown	Pe/ppe	ELISPOT of Rv3429 achieved sensitivities of 47% in aTB and specificities of 93% in BCG-vaccinated HC.	Chen et al., 2009

*^a^These antigens were included in both LTBI and RD.*

*^*b*^The information of Product, Function, and Functional category of each antigen was obtained from mycobrowser database (https://mycobrowser.epfl.ch/) on April 26, 2021.*

*aTB, active TB; CC, TB close contacts (subjects exposed to *M. tuberculosis* for less than three months); DTH, delayed-type hypersensitivity; DosR, Dormancy survival regulon antigens; HCs, health controls; HCWs, healthcare workers (individuals exposed to *M. tuberculosis* at least 2 years); NS, nutrition starvation-associated antigens; PPD, purified protein derivative; PTB, pulmonary TB; QFT, QuantiFERON; RD, Region of Difference; TB, tuberculosis.*

(2) Seven antigens belonging to both NS-associated antigens and RD13 exhibit considerable potential, including Rv2653c, Rv2654c, Rv2656c, Rv2657c, Rv2658c, Rv2659c, and Rv2660c. Among the seven antigens, Rv2654c and Rv2660c were the most studied antigens. A previous study showed that Rv2654c induced a higher IFN-γ level in patients with TB than in healthy controls (HCs) and had a high overall agreement with T-SPOT.TB (98.0%) (Luo et al., 2015). An additional study reported that peptide Rv2654c_51–65_ boosted the quantitative performance of the QFT-GIT assay from 1.83 IU/ml to 2.83 IU/ml (Horvati et al., 2016). Rv2660c is one of the most promising antigens for the differential diagnosis of LTBI. He et al. (2015) found that Rv2660c induced a significantly higher number of IFN-γ-producing cells and levels of cytokines [IFN-γ, IL-2, IL-10, and macrophage inflammatory protein 1-alpha (MIP-1α)] in patients with LTBI than in patients with TB and a higher ratio of IFN-γ^+^ CD4^+^ T cells in the LTBI group than in the aTB or HC group. Moreover, it is important to note that although Rv2660c induced a stronger immune response in LTBI than in aTB, it is a component of vaccine H56:IC31, which affects the differential diagnosis of Rv2660c once the vaccine is available (Luabeya et al., 2015). Rv2653c was used as a diagnostic skin test reagent in comparison with a standard PPD, and it was found that this antigen stimulated less of a delayed-type hypersensitivity (DTH) reaction in *M. tuberculosis*-sensitized guinea pigs, but no response was observed in BCG-vaccinated guinea pigs (Kasempimolporn et al., 2019), suggesting that this antigen can eliminate the interference of BCG vaccination with test results. Our previous study showed that Rv2657c reacted with the sera from LTBI guinea pigs but not those of healthy guinea pigs and that the number of IFN-γ-producing cells induced by Rv2657c and Rv2659c was significantly higher in patients with LTBI than in patients with aTB or HCs (Bai, 2015; Yang, 2018), indicating that Rv2657c and Rv2659c might be promising antigens for the differential diagnosis of LTBI. Additionally, the roles of Rv2656c and Rv2658c in the differential diagnosis of LTBI are unknown.

(3) Ten antigens belong to both LTBI others and RDs, including four antigens in LTBI others-RD1 (Rv3872-Rv3879), three antigens in LTBI others-RD2 (Rv1978, Rv1980c, and Rv1981c), one antigen in LTBI others-RD6 (Rv1511), and two antigens in LTBI others-RD11 (Rv3425 and Rv3429). Among them, the antigen that is most frequently studied is Rv3872. Previous studies have reported that the sensitivity and specificity of Rv3872 for detecting LTBI in children were 76% and 80%, respectively, which could elicit a stronger immunoreactivity for discriminating TB from HCs in patients who have received BCG vaccination (Mukherjee et al., 2007; Mahmoudi et al., 2017). In contrast, the remaining antigens (Rv3879, Rv1978, Rv1980c, Rv1981c, Rv3425, and Rv3429) were only tested between patients with TB and HCs with BCG vaccination or LTBI and HCs with BCG vaccination (Mukherjee et al., 2007; Zhang et al., 2007; Chen et al., 2009; Hinks et al., 2009; Wang et al., 2013; Luo et al., 2017; Mahmoudi et al., 2017; Cao et al., 2021). Hinks et al. (2009) compared the responses to a range of RD1-encoded antigens (ESAT-6, CFP-10, Rv3879c, Rv3878, Rv3873, and Rv2031c) and defined a similar hierarchy of immunodominance for these antigens in both aTB and LTBI subjects: CFP-10 > ESAT-6 > Rv3879c > Rv3878 > Rv3873 > Rv2031c. Furthermore, Rv3425 has been evaluated as a candidate for the development of a vaccine for TB control, which may affect its accuracy in detecting LTBI (Wang et al., 2008).

Our findings suggest that these 21 LTBI-RD-associated antigens are promising alternatives to diagnostic biomarkers for discriminating LTBI from aTB and HCs in patients who have received BCG vaccination. By analyzing the above research results, it is not difficult to find that the intensity of the immune response induced by these LTBI-RD-related antigens was always higher in the LTBI group than in the aTB group. However, the ESAT-6/CFP10 responses, which are not dormancy related, were also much higher in the LTBI group than in the aTB group; thus, the relative advantages of these LTBI-RD-related antigens are unclear. The reason for this may be as follows: the immunity of patients with aTB is weakened and the responses of T cells are usually suppressed (Figure 1A). Therefore, the sensitivity and specificity of these LTBI-RD-related antigens need to be validated in further studies with larger sample sizes and broader population coverage in the future. In addition, there are also problems in the differential diagnosis of LTBI-RD-associated antigens: (1) the immunogenicity of these antigens is significantly lower than that of proliferative antigens; (2) the population coverage rate of the immune responses induced by these antigens is low; (3) most of the *M. tuberculosis* in the macrophages of patients with LTBI are in a state of latent infection, but a small part of them is in a state of irregular proliferation; and (4) not all *M. tuberculosis* in patients with active TB are in a proliferative state and are influenced by the immune state of the host and the treatment of chemotherapy drugs, with some *M. tuberculosis* possibly also being in a latent state.

#### Peptides

The LTBI-RD-associated antigens derived from *M. tuberculosis* constitute a potential source of specific antigens for immunodiagnosis and vaccine development for LTBI. Screening the diagnostic potential of specific T- or B-cell epitopes from LTBI-RD-associated antigens and verifying their peptides in different populations are a new concept for the differential diagnosis of LTBI. Currently, most of the studies on peptides derived from LTBI-RD-related antigens in differentiating LTBI from aTB and HCs are still in their initial stages. Only a few studies have screened immunodominant peptides of latency and/or RD-related antigens in animal models and humans, such as Rv1985c_pool2_, Rv1985c_pool4_, Rv3425_pool1_ (Wang et al., 2013), Rv1733c_57–84_ (Coppola et al., 2015), Rv2654c_51–65_ (Horvati et al., 2016), peptide pools from RD1 antigens (Rv3871 to Rv3878) (Mustafa et al., 2002), T-cell epitopes of Rv3872 and Rv3873 (Hanif et al., 2011; Jiang et al., 2016), and Rv3425_118–126_ (LIASNVAGV) (Chen et al., 2012). However, most of these mapped and identified peptides can only discriminate aTB from HCs with BCG vaccination and fail to distinguish LTBI from aTB.

The limitation of these studies is that they only focused on epitopes singly identified from CD4^+^ T lymphocytes [helper T lymphocytes (Th)] or CD8^+^ T lymphocytes (CTLs) rather than epitopes synchronously identified from Th cells, CTL cells, and B cells. Although the traditional view is that the host clearance and killing of *M. tuberculosis* mainly depend on CD4^+^ T lymphocytes and CD8^+^ T lymphocytes, accumulated data show that B lymphocytes also play an irreplaceable role in fighting against *M. tuberculosis* (Gong et al., 2018, 2021; Watson et al., 2021). Therefore, peptides binding to Th cells, CTL cells, and B cells should be considered when designing new candidate biomarkers based on epitopes for distinguishing LTBI from aTB and BCG-vaccinated HCs.

Furthermore, MHC molecules affect the efficacy of peptide-based vaccines and diagnosis (Gong et al., 2021; Jia et al., 2021). It is well known that recognition peptides produced by CD4^+^ or CD8^+^ T cells are mostly restricted to MHC-II or MHC-I molecules, respectively. Human leukocyte antigens (HLAs) are highly polymorphic; thus, the selection of peptides that can be recognized by multiple HLAs will increase the population coverage of peptide-based diagnosis (Bui et al., 2006). As the frequency of HLA expression varies greatly among different ethnicities, the problem of population coverage related to MHC polymorphisms becomes more complicated. Thus, without careful consideration, peptide-based vaccines or diagnostic candidates will result in ethnically biased population coverage.

### Novel Biomarkers Derived From Hosts

Our previous research and other large amounts of data show that TB is not only an infectious disease but also an immune disease (Gong et al., 2018, 2020, 2021; Li et al., 2020; Liang et al., 2021; Rambaran et al., 2021). The pathogenesis of TB is closely related to the host’s immune response. Therefore, to promote the development of sensitive methods for the detection and treatment of LTBI, it is particularly urgent to understand the host responses involved in the establishment and maintenance of LTBI (Banerjee et al., 2021).

#### Cytokines

Cytokines are small molecular polypeptides or glycoproteins synthesized and secreted by immune cells (such as monocytes, macrophages, T cells, B cells, and NK cells) and some non-immune cells (such as endothelial cells, epidermal cells, and fibroblasts) after stimulation. Cytokines have a variety of biological functions, such as regulation of cell growth, differentiation and maturation, immune response, inflammation, wound healing, tumor growth, and function maintenance. Levels of host cytokines vary in the different stages of *M. tuberculosis* infection, which may serve as biomarkers for differentiating LTBI from aTB (Luo et al., 2019).

Detection of IFN-γ stimulated by specific antigens of *M. tuberculosis* is a common practice in most IGRA experiments. Won et al. (2017) measured the levels of 29 cytokines in 48 patients with aTB, 15 LTBI, and 13 HCs and found that eight cytokines [GM-CSF, IFN-γ, IL-1RA, IL-2, IL-3, IL-13, interferon-gamma-inducible protein 10 (IP-10), and MIP-1β] in the TB-infected group were significantly different from those of the HCs, and that eight cytokines [EGF, GM-CSF, IFN-γ, IL-2, IL-5, IL-10, TNF-α, and vascular endothelial growth factor (VEGF)] were significantly different between aTB and LTBI. However, the limitation of this study is its small sample size, with its results needing to be further verified through another study with a large sample size. In 2018, Meier et al. (2018) performed a systematic review to summarize the cytokines measured in 34 included studies, with as many as 26 cytokines used as biomarkers in these studies, including IFN-γ, TNF-α, IL-2, IL-10, IL-17, IL-8, IL-6, IP-10, IL-1β, monocyte chemoattractant protein-1 (MCP-1), IL-12, IL-12p40, MCP-2, fractalkine, granzyme B, GM-CSF, IFN-α2, sIL-2Ra, IL-4, IL-5, IL-13, MIP-1β, Regulated upon Activation, Normal T cell Expressed and presumably Secreted (RANTES), sCD40L, TGF-α, and VEGF. Among these 26 cytokines, the top 5 cytokines used as biomarkers for discriminating between LTBI and patients with aTB were IFN-γ (34 publications), TNF-α (15 publications), IL-2 (12 publications), IL-10 (10 publications), and IL-17 (9 publications) (Supplementary Figure 1).

**FIGURE 3:**
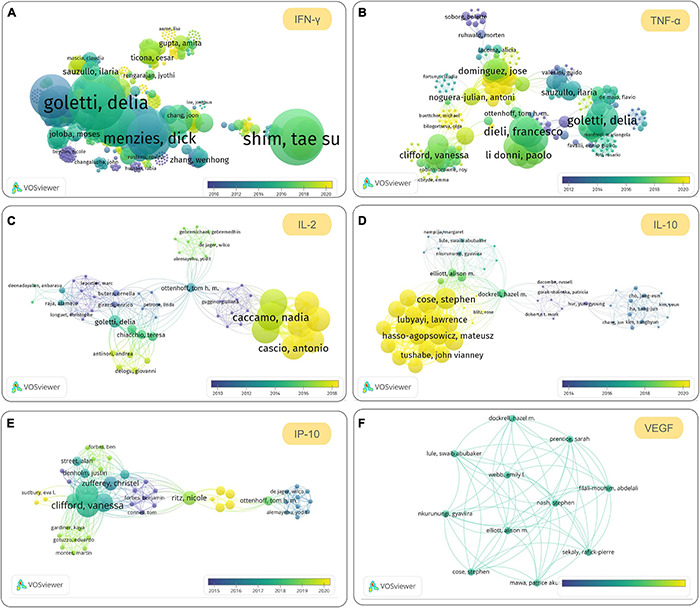
The most promising cytokines to be used to differentiate and diagnose latent tuberculosis infection (LTBI) in the future. The co-authorship maps of interferon (IFN)-γ **(A)**, tumor necrosis factor (TNF)-α **(B)**, interleukin (IL)-2 **(C)**, IL-10 **(D)**, interferon-gamma-inducible protein 10 (IP-10) **(E)**, and vascular endothelial growth factor (VEGF) **(F)** cytokines based on bibliographic data obtained from Web of Science were visualized by using VOSviewer version 1.6.16 (developed by Nees Jan van Eck and Ludo Waltman at Leiden University’s Centre for Science and Technology Studies CWTS). The search terms used in Web of Science are “LTBI” and cytokine name (topic terms), the Timespan was set from 1900 to 2021, the database was set “All databases,” and other parameters are default values. The parameters in VOSviewer were set as below: Type of analysis, Co-authorship; Unit of analysis, Authors; Counting method, Full counting; Normalization method, Association strength; Layout, Attractions 2, Repulsion-1; Clustering, Resolution 1.00, Min. cluster size 1; other parameters are default values.

Although IFN-γ and TNF-α are still the most popular cytokines in the differential diagnosis of LTBI, studies on IL-2, IL-10, IP-10, and VEGF have been increasing in recent years. To understand the latest research progress on these cytokines for discriminating between LTBI and aTB, we searched publications related to them in the Web of Science database. We found that the number of publications related to IFN-γ, TNF-α, IL-2, IL-10, IP-10, and VEGF were 1,419, 418, 136, 62, 96, and 18, respectively (Figure 3). The co-authorship map of IFN-γ (Figure 3A), TNF-α (Figure 3B), IL-2 (Figure 3C), IL-10 (Figure 3D), IP-10 (Figure 3E), and VEGF (Figure 3F) cytokines based on bibliographic data showed similar trends and popularity to the systematic review. In 2020, two meta-analyses were conducted to evaluate candidate cytokines as biomarkers to distinguish LTBI infection from aTB. A systematic meta-analysis by Sudbury et al. (2020) measured 100 cytokines among 56 included studies and found that the most frequently studied cytokines were IFN-γ, IL-2, TNF-α, IP-10, IL-10, and IL-13. A meta-analysis by Qiu et al. (2020) evaluated seven cytokines, including IFN-γ, TNF-α, IP-10, IL-2, IL-10, IL-12, and VEGF, suggesting that cytokines IL-2, IFN-γ, and VEGF can be utilized as promising biomarkers to distinguish LTBI from aTB. Furthermore, compared to IFN-γ, the level of IP-10 was higher and was released independently of age, suggesting that IP-10 may be a candidate biomarker in the pediatric population (Alsleben et al., 2012).

Although individual cytokines provide considerable potential for use in the differential diagnosis of LTBI, for most cytokines, findings were heterogeneous among studies. These differences in results or even diametrically opposite conclusions may be related to differences in study design, experimental methods, statistical methods, individual genetic backgrounds, antigen types, sample sizes, and environmental factors (Rambaran et al., 2021). Therefore, reducing or even eliminating the heterogeneity between different research results is a limitation that needs to be overcome. Recent studies have found that a combination of cytokines may be an alternative to solve this problem. Wang et al. (2018) identified a six-cytokine biosignature (including IFN-γ, IL-10, IL-1Ra, IP-10, VEGF, and IL-12p70) to discriminate between aTB and LTBI, resulting in a sensitivity of 88.2% and a specificity of 92.1% in a biomarker validation cohort (*n* = 216) and a sensitivity of 85.7% and a specificity of 91.3% in a prospectively recruited clinical validation cohort (*n* = 194), respectively. Coincidentally, Korma et al. (2020) evaluated the sensitivity, specificity, and accuracy of the combination of seven cytokines (IFN-γ, IP-10, IL-2R, C-X-C Motif Chemokine Ligand 9 (CXCL-9), IL-10, IL-4, and TNF-α) based on gene expression in differentiating between aTB and LTBI. They demonstrated that the combination of IL-10, IP-10, and IL-4 could differentiate pulmonary patients with TB from latent patients with TB with a sensitivity and specificity of 77.1% and 88.1%, respectively (Korma et al., 2020). Luo et al. (2019) identified potential biomarkers from 38 plasma cytokines to discriminate among the different stages of *M. tuberculosis* infection in its study participants, who were composed of 78 patients with aTB, 73 patients with LTBI, and 76 HCs. They found that the combination of five cytokines (IP-10, MCP-1, IL-1α, IL-10, and TNF-α) had an excellent performance in diagnosing LTBI, with 94% sensitivity and 81.25% specificity, and that the combination of three cytokines (eotaxin, macrophage-derived chemokine (MDC), and MCP-1) had a 0.94 area under the curve (AUC) in differentiating ATB from LTBI with 87.76% sensitivity and 91.84% specificity, respectively (Luo et al., 2019). The above data show that the combination of multiple cytokines can significantly improve specificity and sensitivity, which also points out the direction of future research.

#### Antibodies

Antibody responses are a major form of defense against microbes (Vinuesa et al., 2016). *M. tuberculosis* infection has been reported to induce antibody responses, but it is still unclear whether patients with aTB or those with LTBI produce protective antibodies, as well as which antigens these target (Feng et al., 2013; Loxton and van Rensburg, 2021). With the emerging evidence regarding immune modulation, the complete characterization of B cells and humoral immunity could be of significant value (Dubois Cauwelaert et al., 2016). In 2013, Feng et al. (2013) developed a novel 38F-64F indirect ELISA (including 38 kDa, ESAT-6, CFP10, Mtb8.4, MPT64, TB16.3, and Mtb8) method to detect TB and LTBI, and the results showed that the sensitivity of the 38F-64F indirect ELISA was much higher than that of the sputum culture test (86.91% vs. 50.62%), with the sensitivity of the sputum smear test (78.64% vs. 47.57%) accounting for 74.16% and 37.14% of patients with TB and patients with LTBI, respectively. Five years later, a study screened serum biomarkers of 40 serum samples from patients with LTBI and aTB using a proteome microarray containing 4,262 antigens, and the results suggested that specific IgG levels of 152 *M. tuberculosis* antigens were significantly higher in patients with aTB than in LTBI (Cao et al., 2018). However, any single antigen-specific antibody is not enough to be used to cover the antibody profiles of all patients with TB. Therefore, the combination of antigen-specific antibodies may be an alternative for increasing sensitivity and specificity. Yan et al. (2018) reported that the sensitivity and specificity of individual antigen-specific antibodies are relatively low in detecting patients with TB not infected with the bacteria, but the combination of six antigen-specific antibodies (LAM, 38KD, MPT32, EspC, MPT64, and Mtb81) increased sensitivity and specificity to 69.6% and 77.0%, respectively. Similarly, another study also demonstrated that the sensitivity (96.6%) and specificity (92.3%) of IgA/IgG of a fused MT10.3:MPT64 protein were significantly higher than those of single antigens in pleural TB cases (da Silva et al., 2019). These results suggest that combining multiple antigen-specific antibodies can significantly improve the sensitivity and specificity of a single antigen-specific antibody, which will help in the diagnosis of TB and LTBI, but the current data are insufficient and further research is needed.

#### Markers of Immune Cells

As mentioned above, TB is a heterogeneous disease with a wide range of infectious and immunological profiles. It is well-known that the immunological markers of T-lymphocyte subsets and NK cell surfaces correlate with cell differentiation, latency/disease status, and outcome, making these cell surface markers potential targets for TB diagnosis and treatment (Petruccioli et al., 2016).

CD4^+^ T lymphocytes play a fundamental role in controlling *M. tuberculosis* infection, and the biomarker expression profiles of CD4^+^ T cell surfaces are associated with bacterial loads at different infection stages. Assessment of CD27 and/or CCR4 expression on CD4^+^ T cells has been reported to be a robust biomarker for discriminating among TB stages. A previous study analyzed the expression of CD27 and CCR4 biomarkers in IFN-γ^+^CD4^+^ T cells collected from patients with aTB and patients with LTBI and found that the proportion of CD27^–^IFN-γ^+^CD4^+^ T cells or CCR4^+^ IFN-γ^+^CD4^+^ T cells was significantly higher in patients with aTB than in those with LTBI in response to PPD or ESAT-6/CFP-10 recombinant proteins and that the proportion of CD27^–^CCR4^+^IFN-γ^+^CD4^+^ T cells was significantly associated with aTB. Furthermore, other studies also found that the CD38^+^CD27^–^TNF-α^+^CD4^+^ T-cell subset is a potential biomarker for diagnosing TB with 96.15% specificity and 90.16% sensitivity (Acharya et al., 2021), with IFN-γ^+^CD27^low^CD4^+^ T cells described as aTB biomarkers (Latorre et al., 2018). These findings suggest that the loss of CD27 and the increase in CD38 and CCR4 biomarkers could be associated with uncontrolled *M. tuberculosis* infection. In addition, innate T cells, such as MAIT and invariant NK T (iNKT) cells, play roles in recognizing *M. tuberculosis*-infected cells. Accumulating evidence highlights the importance of both MAIT and iNKT cells in controlling TB infection (Ruibal et al., 2021). It has been reported that the number of both cell types was significantly higher in subjects with LTBI than in patients with aTB or uninfected individuals, and that iNKT cells from patients with LTBI showed lower PD-1 expression than those from patients with aTB (Paquin-Proulx et al., 2018).

### New Models and Algorithms

As mentioned above, the immunological methods that are frequently used, such as skin tests and IGRAs, are intrinsically unable to discriminate LTBI from aTB. Recently, with the development of artificial intelligence (AI) and bioinformatics, new strategies have been introduced to improve diagnostic performance in distinguishing LTBI from aTB, such as the ImmunoScore (IS) model, Cox proportional hazards model, expectation maximization algorithm, silico mapping algorithm, and random forest algorithm (Villate et al., 2006; Zhou et al., 2017; Ndzi et al., 2019; Wu et al., 2019; Rambaran et al., 2021). IS is a novel and promising prognostic tool that is widely used in tumors. It has been reported that the IS model can effectively distinguish aTB from LTBI, with a sensitivity of 95.7% and a specificity of 92.1% (Zhou et al., 2017). The Cox proportional hazards model was applied to estimate the survival experience and risk factors among patients with TB, and these studies observed that associations were stronger in the Cox proportional hazards model, suggesting that the impact of behavioral and clinical variables affected TB outcome (Tolosie and Sharma, 2014; Abedi et al., 2019; Rambaran et al., 2021). Villate et al. (2006) analyzed infection data of LTBI and *M. avium* using the expectation maximization algorithm, and their results confirmed that the mixture models using the expectation maximization algorithm can better estimate the probability of LTBI in the presence of other NTM and BCG vaccinations. Ndzi et al. (2019) developed a silico mapping algorithm to discriminate LTBI from aTB and found that people with the QFT-GIT^+^ and DRB1^∗^10-DQB1^∗^02 haplotype^+^ may have a higher chance of developing LTBI and that those with the QFT-GIT^+^ and DRB1^∗^10-DQB1^∗^02 haplotype^–^ may progress to aTB. Wu et al. (2019) also developed a random forest algorithm to discriminate between aTB and LTBI based on the test data of T-SPOT.TB, and the results indicated that the sensitivity and specificity of this algorithm were 82.87% and 88.03%, respectively, which were higher than those of T-SPOT.TB (82.80% and 70.00%, respectively), TB (82.80% and 74.20%), and QFT-GIT (71.40% and 81.00%). Although these innovative strategies are still in their initial development stages with only a few studies conducted on them, they still provide valuable information for a comprehensive understanding of TB and allow for new insights on differential diagnosis of LTBI and aTB to be gained.

### Omics Technologies

In recent years, advances in multiplexing and information technology have enabled researchers to perform more comprehensive analyses of complete genomes, transcriptomes, proteomes, and metabolomes. The use of these omics techniques has provided valuable insights for understanding the transcriptomic, proteomic, and metabolomic changes of *M. tuberculosis* during dormancy and reactivation.

#### Transcriptomics

The complete genome sequencing of the *M. tuberculosis* H37Rv strain in 1998 allowed for improvement in our understanding of *M. tuberculosis* (Cole et al., 1998). Transcriptome analysis provides insights into the expression profiles of genes in human samples (such as blood) during the different stages of *M. tuberculosis* infection. As early as 2007, Jacobsen et al. (2007) compared the gene expression signature of peripheral blood mononuclear cells (PBMCs) collected from patients with aTB, individuals with LTBI, and HCs by microarray analysis and found that three genes (*Ras-associated GTPase 33A, lactoferrin*, and *CD64*) were sufficient for classifying these three groups of individuals. Almost at the same time, Mistry et al. (2007) expanded the application of this technology to patients with aTB and individuals with LTBI in patients with recurrent disease and cured patients with TB using DNA array technology and quantitative reverse-transcriptase polymerase chain reaction (qRT-PCR). Their results demonstrated that a set of nine genes could discriminate the four groups of individuals, including *ATP5G1*, *ASNA1*, *C14orf2*, *KIAA2013*, *LY6G6D*, *NOLA3*, *RIN3*, *SOCS3*, and *TEX264*. Zhang et al. (2007) determined the transcriptomic profiles of exosomal RNAs collected from 30 HCs, 30 patients with aTB, and 30 individuals with LTBI. In comparison with the HCs, five genes were significantly upregulated and highly expressed in individuals with LTBI, but they were downregulated and expressed at low levels in patients with aTB. These included *TTLL10*, *RP11-358N4.3*, *RMRP*, *PPPH1*, and *RN7SK* (Lv et al., 2017), which represent novel biomarkers for the diagnosis of LTBI and aTB. More recently, Bayaa et al. (2021) conducted a multicenter prospective nested case-control study to evaluate the performance of a human blood transcriptomic signature termed *RISK6* in the diagnosis of LTBI and aTB, and their results showed that the sensitivity and specificity of *RISK6* for discriminating aTB from LTBI were 90.9% and 88.5%, respectively.

Transcriptomics is also used to assess the risk of LTBI developing into aTB. In 2010, a study analyzed the genome-wide transcriptional profiles of blood samples obtained from patients with aTB, individuals with LTBI, and HCs and found that the signatures of aTB were observed in 10%–20% of patients with LTBI, indicating that these identified individuals with LTBI were at risk of developing aTB (Berry et al., 2010).

#### Proteomics

Proteomics, developed on the basis of genomics, is complementary to studies at the transcriptional level (Betts, 2002). The emergence and development of proteomics have enabled the discovery of new biomarkers at the gene level, which can be used to distinguish LTBI from aTB to be verified at the protein level. Previous studies have identified a large number of biomarkers that are differentially regulated during dormancy and reactivation using proteomics and transcriptomics; for example, the DosR and genes involved in DNA replication are upregulated and downregulated, respectively, during dormancy, but undergo the opposite changes during reactivation (Kundu and Basu, 2021). Cao et al. (2018) performed a proteome microarray assay containing 4,262 antigens to identify potential serum biomarkers for distinguishing between LTBI and aTB, and their results indicated that antigens Rv2031c, Rv1408, and Rv2421c had higher AUCs of 0.8520, 0.8152, and 0.7970, respectively. With the development of high-resolution quantitative proteomics and label-free quantitative proteomics, a growing number of protein signatures that can be used for the differential diagnosis of LTBI and aTB have been discovered. Mateos et al. (2019) performed high-resolution quantitative proteomics using an LTQ-Orbitrap-Elite platform to screen for potential protein signatures in the sputum and saliva of patients with aTB, individuals with LTBI, and HCs. Their findings suggested that a reduction in proteins related to glucose and lipid metabolism was observed in the saliva of patients with TB, while the proteomic signatures of sputum from individuals with LTBI and HCs consisted of proteins related to bitterness perception, pathogen defense, and the innate immune response. Similarly, Sun et al. (2018) performed label-free quantitative proteomics to determine plasma signatures for discriminating PTB from LTBI or HCs and found that a combination of alpha-1-antichymotrypsin (ACT), alpha-1-acid glycoprotein 1 (AGP1), and E-cadherin (CDH1) showed 81.2% sensitivity and 95.2% specificity in discriminating PTB from LTBI and 81.2% sensitivity and 90.1% specificity in discriminating PTB from HCs.

#### Metabolomics

Metabolomics is a field newly developed after genomics and proteomics, and it is an important part of systems biology. Genomics and proteomics explore the processes of life at the gene and protein levels, respectively. In fact, many life activities in cells occur at the level of metabolites, such as cell signaling, energy transfer, and cell-to-cell communication (Griffiths and Wang, 2009). Since the metabolome is the final downstream product of the genome, transcriptome, and proteome, any interference at these levels will change the metabolome. In 2017, Preez et al. (2017) reviewed and summarized abundant biomarkers related to TB diseases that have been identified in culture (Phillips et al., 2007; Syhre and Chambers, 2008; Olivier and Loots du, 2012; Lau et al., 2015), human sputum (Schoeman et al., 2012; du Preez and Loots, 2013), animal or human blood and tissue (Shin et al., 2011; Somashekar et al., 2012; Weiner et al., 2012; Che et al., 2013; Zhou et al., 2013; Frediani et al., 2014; Feng et al., 2015), human urine (Banday et al., 2011; Das et al., 2015), and human breath (Phillips et al., 2007; Syhre et al., 2009; Phillips et al., 2010; Kolk et al., 2012) using a metabolomics research approach (Preez et al., 2017). However, most of the signatures identified in these studies were obtained by comparing patients with TB and HCs or other diseases, and only one study was performed among patients with TB, individuals with LTBI, and HCs. In this study, Weiner et al. (2012) explored the metabolites in serum collected from three groups of individuals, and their results showed that compared to individuals with LTBI, patients with aTB showed increased indoleamine 2,3 dioxygenase 1 activity, decreased phospholipase activity, increased adenosine metabolite abundance, and increased levels of fibrotic disease indicators. Furthermore, they also found that the abundance of inosine was significantly lower in individuals with LTBI than in patients with TB and HCs (Weiner et al., 2012). More recently, Dutta et al. (2020) analyzed the plasma metabolite profiles of 16 aTB children and 32 household contacts in India using liquid chromatography-mass spectrometry and identified three metabolites (N-acetylneuraminate, quinolinate, and pyridoxate) that could correctly discriminate TB status at distinct times during treatment, with AUCs of 0.66, 0.87, and 0.86, respectively. Although the above studies have used metabolomics techniques to identify abundant biomarkers for distinguishing aTB from individuals with LTBI, they have not linked metabolomics with transcriptomics and proteomics. Integrating metabolomics data with transcriptomics and proteomics data to comprehensively analyze and identify novel biomarkers is a direction for future omics technology development. Saiboonjan et al. (2021) investigated the transcriptomics, proteomics, and metabolomics profiles of three strains of *M. tuberculosis* (including drug-susceptible Beijing strain, multidrug-resistant Beijing strain, and H37Rv strain) cultured under stress conditions to mimic the environment of the host granuloma. They found that NarJ (respiratory nitrate reductase delta chain) was significantly upregulated at the protein and mRNA levels in all three *M. tuberculosis* strains and that NarJ plays an important role in nitrate metabolism during the adaptation of *M. tuberculosis* to stressful and intracellular environments and subsequent establishment of LTBI (Saiboonjan et al., 2021).

### Microbiota

Hundreds of millions of microorganisms have coexisted with humans in the respiratory tract and gastrointestinal tract for thousands of years. However, research on them has been recently increasing. A growing number of studies have suggested that the microbiota plays an essential role in maintaining normal health, developing the immune system, and providing protection against pathogens (Khan et al., 2016; Dumas et al., 2018; Gupta et al., 2018; Liu et al., 2021). However, the impact of microbiota on host defense against *M. tuberculosis* infection is poorly understood. A recent review published in PLOS Pathogens summarized 14 microbiome studies performed on animal models of TB and patients with TB (Mori et al., 2021). Among these studies, nine focused on the relationship between microbiota in the intestinal tract and *M. tuberculosis*, including five studies on patients with TB, three studies on mouse models, and one study on rhesus macaques. Compared with the number of studies on the intestinal tract, only six studies have been performed to explore the correlations between microbiota in the respiratory tract and *M. tuberculosis*, including five studies on patients with TB and one study on rhesus macaques. These studies investigated the impact of *M. tuberculosis* infection on the host microbiome, and all of them found that *M. tuberculosis* infection changed the composition of microbiota, with only four studies reporting the impact of microbiota changes caused by *M. tuberculosis* infection on the immune system. It is important to note that one study compared the changes in the composition of microbiota and its effects on the immune system among patients with TB (*n* = 25), patients with LTBI (*n* = 32), and healthy individuals (*n* = 23) (Huang et al., 2019). The results suggested that dysbiosis with a higher abundance of Bacteroidetes and a low ratio of Firmicutes to Bacteroidetes was related to systemic proinflammation in patients with aTB, while the abundance of Coriobacteriaceae was positively related to the level of IFN-γ and CD4^+^ T cells in LTBI.

It is becoming increasingly recognized that the composition of the respiratory and gastrointestinal microbiota may affect the systemic immune responses of the host, and that microbiome–immune interactions may affect the susceptibility, manifestation, and progression of *M. tuberculosis* infection (Wipperman et al., 2021). However, the results of most studies are inconsistent and contradictory. This may be due to differences in species, heterogeneity of microbiota between individuals, sample size, detection methods, and experimental design, among others. The current data cannot determine whether microbiota can be used to distinguish between LTBI and aTB, and more high-quality studies with larger sample sizes are urgently needed to clarify this.

## Conclusion

Tuberculosis is a global infectious disease that threatens human health, and LTBI is the biggest obstacle to achieving the goal of ending the global TB epidemic shared by the UN Sustainable Development Goals and WHO’s End TB Strategy. As an ancient test that has been used to diagnose TB for more than 100 years, the TST has made great contributions to the control of TB. However, the TST has several challenges, such as the need for return visits, low specificity and sensitivity, false positives with BCG vaccination, cross-reactivity with NTMs, and false positives in immunosuppression and deficiency. To overcome these shortcomings of the TST, several new detection methods have been developed, such as the Diaskintest, C-Tb skin test, EC-Test, and T-SPOT.TB, QFT-GIT, QFT-Plus, LIAISON QFT-Plus, and LIOFeron TB/LTBI. The application of RD antigens (ESAT-6, CFP-10, TB7.7, and Ala-DH) prevents these novel methods from cross-reacting with BCG inoculation and most NTM infections, with the additional advantages of having higher specificity and sensitivity.

However, these methods cannot distinguish between LTBI and aTB. To investigate the reasons behind this, we believe that the abovementioned methods (1) were only designed based on RD antigens rather than LTBI-RD-associated antigens (Rv1736c, Rv1737c, Rv2031c, Rv2626c, Rv2653c-Rv2660c, Rv1511, Rv1978, Rv1980c, Rv1981c, Rv3872, Rv3873, Rv3878, Rv3879c, Rv3425, and Rv3429), without considering the difference in profiles of antigen expression between latent and active infection of *M. tuberculosis*; (2) only detect the number of IFN-γ-producing T cells and serum concentrations of IFN-γ produced by CD4^+^ and/or CD8^+^ T cells, but not of other biomarkers, such as antigen-specific antibodies, IL-2, IL-10, IP-10, and VEGF, nor their combinations; and (3) did not use the latest models and algorithms to improve their diagnosis performance for distinguishing between LTBI and aTB.

Thus, areas for further investigation and development should include (1) understanding the differential expression of various antigens in latent and active infection of *M. tuberculosis* based on omics technologies and studies with larger sample sizes, (2) focusing on multiple cytokines and/or antibodies derived from hosts that have been identified by previous studies, (3) improving the sensitivity and specificity of LTBI differential diagnosis using new models, algorithms, and IS, and (4) exploring the differences in microbiota between patients with LTBI and aTB to find new evidence for improving the diagnostic accuracy of LTBI and aTB.

## Author Contributions

XW and WG did the conceptualization and wrote–reviewed and edited. WG did the data curation, formal analysis, funding acquisition, methodology, software, and writing–original draft. Both authors contributed to the article and approved the submitted version.

## Conflict of Interest

The authors declare that the research was conducted in the absence of any commercial or financial relationships that could be construed as a potential conflict of interest.

## Publisher’s Note

All claims expressed in this article are solely those of the authors and do not necessarily represent those of their affiliated organizations, or those of the publisher, the editors and the reviewers. Any product that may be evaluated in this article, or claim that may be made by its manufacturer, is not guaranteed or endorsed by the publisher.

## Supplementary Material

Supplementary Figure 1 Twenty-six cytokines were summarized from 34 included publications by Meier, N. R. et al. in a systematic review (Meier et al., 2018). The number of publications related each cytokine was showed as histogram.

## Abbreviations

Ala-DH: alanine dehydrogenase
ARI: annual risk in infection
aTB: active tuberculosis
AUCs: areas under the curve
BCG: Bacillus Calmette–Guérin
CFP-10: culture filtrate protein 10
CLIA: chemiluminescent immunoassay
CTL: cytotoxic T lymphocytes
DCs: dendritic cells
DC-SIGN: DC-specific intercellular adhesion molecule-3 grabbing nonintegrin
DosRs: dormancy survival regulon antigens
EGF: epidermal growth factor
ELISA: enzyme-linked immunosorbent assay
ELISpot: enzyme-linked immunospot
ESAT-6: early secreted antigenic target 6
GM-CSF: granulocyte macrophage colony-stimulating factor
HCs: health controls
HCWs: healthcare workers
HLAs: human leukocyte antigens
IFN- γ: interferon- γ
IGRAs: interferon-gamma release assays
IL: interleukin
IP-10: interferon-gamma-inducible protein 10
IS: ImmunoScore
LTBI: latent tuberculosis infection
MIP-1 β: macrophage inflammatory protein 1 β
NS: nutrition starvation
NTM: non-tuberculous mycobacteria
OT: old tuberculin
PPD: purified protein derivative
PPRs: pattern recognition receptors
QFT-GIT: QuantiFERON-TB Gold In-Tube
QFT-Plus: QuantiFERON-TB Gold-Plus
RAs: reactivation antigens
RD: region of difference
RPF: resuscitation-promoting factor
TAS: toxin–antitoxin system-associated antigens
TB: tuberculosis
Th: helper T lymphocytes
TLR: toll-like receptor
TNF- α: tumor necrosis factor- α
Tregs: regulatory T cells
T-SPOT.TB: T-cell spot of TB assay
TST: tuberculin skin test
VEGF: vascular endothelial growth factor
WHO: World Health Organization.
