# Supplementary figures and images for: Differential Diagnosis of Latent Tuberculosis Infection and Active Tuberculosis: A Key to a Successful Tuberculosis Control Strategy
Source: Front Microbiol. 2021 Oct 22;12:745592. doi: 10.3389/fmicb.2021.745592 (PMC8570039; doi:10.3389/fmicb.2021.745592)

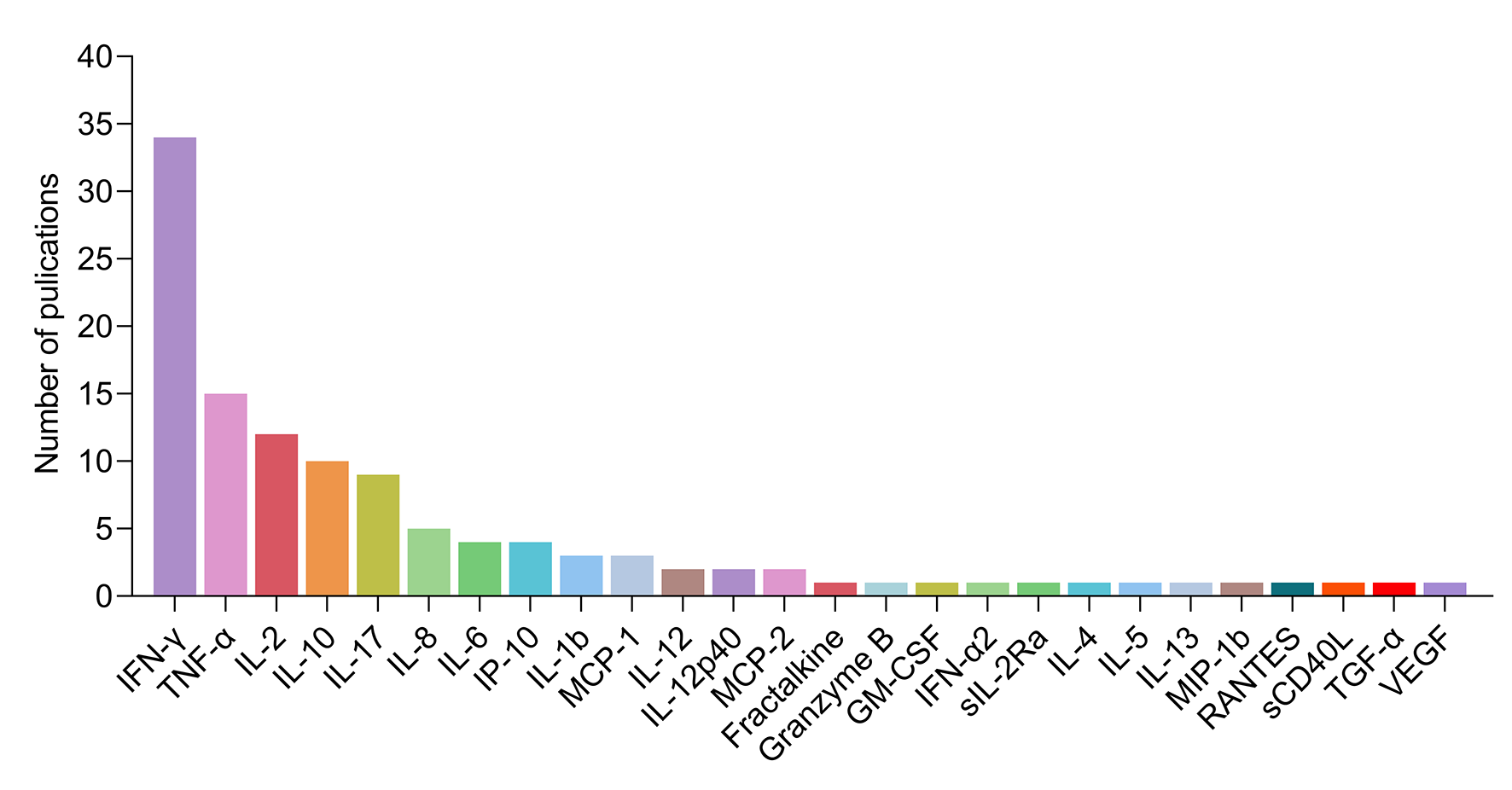

Supplement: Supplementary Figure 1 — Twenty-six cytokines were summarized from 34 included publications by Meier, N. R. et al. in a systematic review (Meier et al., 2018). The number of publications related each cytokine was showed as histogram. [file Image_1.TIF]
