# Supplementary material for: Differential Diagnosis of Latent Tuberculosis Infection and Active Tuberculosis: A Key to a Successful Tuberculosis Control Strategy
Source: Front Microbiol. 2021 Oct 22;12:745592. doi: 10.3389/fmicb.2021.745592 (PMC8570039; doi:10.3389/fmicb.2021.745592)
Supplement: Supplementary file 2 [file Table_1.DOCX]

**Table S1 List of RD-associated antigens**

| **RD No** | | **Antigen name** | | **RD No** | | **Antigen name** | | **RD No** | | **Antigen name** | | | **RD No** | **Antigen name** | | **RD No** | **Antigen name** |
| --- | --- | --- | --- | --- | --- | --- | --- | --- | --- | --- | --- | --- | --- | --- | --- | --- | --- |
| RD1 | Rv3871 | | RD3 | | Rv1580c | | RD7 | | Rv2347c | | RD12 | Rv2073c | | | RD15 | Rv1963c | |
|  | Rv3872 | |  |  | Rv1581c | |  |  | Rv2348c | |  | Rv2074 | | |  | Rv1964 | |
|  | Rv3873 | |  |  | Rv1582c | |  |  | Rv2349c | |  | Rv2075c | | |  | Rv1965 | |
|  | Rv3874 | |  |  | Rv1583c | |  |  | Rv2350c | | RD13 | Rv2645 | | |  | Rv1966 | |
|  | Rv3875 | |  |  | Rv1584c | |  |  | Rv2351c | |  | Rv2646 | | |  | Rv1967 | |
|  | Rv3876 | |  |  | Rv1585c | |  |  | Rv2352c | |  | Rv2647 | | |  | Rv1968 | |
|  | Rv3877 | |  |  | Rv1586c | |  |  | Rv2353c | |  | Rv2648 | | |  | Rv1969 | |
|  | Rv3878 | | RD4 | | Rv0221 | | RD8 | | Rv0309 | |  | Rv2649 | | |  | Rv1970 | |
|  | Rv3879c | |  |  | Rv0222 | |  |  | Rv0310c | |  | Rv2650c | | |  | Rv1971 | |
| RD2 | Rv1978 | |  |  | Rv0223c | |  |  | Rv0311 | |  | Rv2651c | | |  | Rv1972 | |
|  | Rv1979c | | RD5 | | Rv3117 | |  |  | Rv0312 | |  | Rv2652c | | |  | Rv1973 | |
|  | Rv1980c | |  |  | Rv3118 | | RD9 | | Rv3617 | |  | Rv2653c | | |  | Rv1974 | |
|  | Rv1981c | |  |  | Rv3119 | |  |  | Rv3618 | |  | Rv2654c | | |  | Rv1975 | |
|  | Rv1982c | |  |  | Rv3120 | |  |  | Rv3619c | |  | Rv2655c | | |  | Rv1976c | |
|  | Rv1983 | |  |  | Rv3121 | |  |  | Rv3620c | |  | Rv2656c | | |  | Rv1977 | |
|  | Rv1984c | | RD6 | | Rv1506c | |  |  | Rv3621c | |  | Rv2657c | | | RD16 | Rv3400 | |
|  | Rv1985c | |  |  | Rv1507c | |  |  | Rv3622c | |  | Rv2658c | | |  | Rv3401 | |
|  | Rv1986 | |  |  | Rv1508c | |  |  | Rv3623 | |  | Rv2659c | | |  | Rv3402c | |
|  | Rv1987 | |  |  | Rv1509 | | RD10 | | Rv1255c | |  | Rv2660c | | |  | Rv3403c | |
|  | Rv1988 | |  |  | Rv1510 | |  |  | Rv1256c | | RD14 | Rv1766 | | |  | Rv3404c | |
| RD3 | Rv1573 | |  |  | Rv1511 | |  |  | Rv1257c | |  | Rv1767 | | |  | Rv3405c | |
|  | Rv1574 | |  |  | Rv1512 | | RD11 | | Rv3425 | |  | Rv1768 | | | Others* | Rv1737c^1,2^ | |
|  | Rv1575 | |  |  | Rv1513 | |  |  | Rv3426 | |  | Rv1769 | | |  | Rv1736c^1^ | |
|  | Rv1576c | |  |  | Rv1514c | |  |  | Rv3427c | |  | Rv1770 | | |  | Rv2031c^2,3^ | |
|  | Rv1577c | |  |  | Rv1515c | |  |  | Rv3428c | |  | Rv1771 | | |  | Rv2626c^2,4^ | |
|  | Rv1578c | |  |  | Rv1516c | |  |  | Rv3429 | |  | Rv1772 | | |  |  | |
|  | Rv1579c | | RD7 | | Rv2346c | | RD12 | | Rv2072c | |  | Rv1773c | | |  |  | |

*, These antigens were not included in RD, but the previous studies have proved that they are significant different between BCG strain and M. tuberculosis strain.

1, Honaker R W, Stewart A, Schittone S, et al. Mycobacterium bovis BCG Vaccine Strains Lack narK2 and narX Induction and Exhibit Altered Phenotypes during Dormancy[J]. Infection & Immunity, 2008, 76(6):2587-93.

2, Ji P, Fan X, Wu K, Lu S. [Research progress on the antigens associated with latent infection of Mycobacterium tuberculosis]. Chinese Journal of Microbiology and Immunology. 2015;35:59-64. (吉萍, 范小勇, 吴康,等. 结核分枝杆菌潜伏性感染相关抗原的研究进展[J]. 中华微生物学和免疫学杂志, 2015(1):59-65.)

3, Geluk A, Lin MY, van Meijgaarden KE, Leyten EM, Franken KL, Ottenhoff TH, et al. T-cell recognition of the HspX protein of Mycobacterium tuberculosis correlates with latent M. tuberculosis infection but not with M. bovis BCG vaccination. Infection and immunity. 2007;75:2914-21.

4, Pena D, Rovetta AI, Hernandez Del Pino RE, Amiano NO, Pasquinelli V, Pellegrini JM, et al. A Mycobacterium tuberculosis Dormancy Antigen Differentiates Latently Infected Bacillus Calmette-Guerin-vaccinated Individuals. EBioMedicine. 2015;2:884-90.
