# Supplementary material for: Differential Diagnosis of Latent Tuberculosis Infection and Active Tuberculosis: A Key to a Successful Tuberculosis Control Strategy
Source: Front Microbiol. 2021 Oct 22;12:745592. doi: 10.3389/fmicb.2021.745592 (PMC8570039; doi:10.3389/fmicb.2021.745592)
Supplement: Supplementary file 3 [file Table_2.DOCX]

**Table S2 List of latency-associated antigens**

| **Type** | | **Antigen name** | **Type** | | | **Antigen name** | **Type** | | | **Antigen name** | **Type** | | **Antigen name** | | **Type** | | | **Antigen name** |
| --- | --- | --- | --- | --- | --- | --- | --- | --- | --- | --- | --- | --- | --- | --- | --- | --- | --- | --- |
| DosR | Rv0079 | | |  | Rv2003c | | |  | Rv3131 | | | RPF | | Rv0867c | |  | Rv0978c | |

|  | Rv0080 |  | Rv2004c |  | Rv3132c |  | Rv1009 |  | Rv1196 |
| --- | --- | --- | --- | --- | --- | --- | --- | --- | --- |
|  | Rv0081 | DosR | Rv2005c |  | Rv3133c |  | Rv1884c |  | Rv1284 |
|  | Rv0195 |  | Rv2006 |  | Rv3134c |  | Rv2389c |  | Rv1511 |
|  | Rv0386 |  | Rv2007c | RA | Rv0140 |  | Rv2450c |  | Rv1626 |
|  | Rv0491 |  | Rv2028c |  | Rv0246 | NS^1^ | Rv2653c |  | Rv1860 |
|  | Rv0569 |  | Rv2029c |  | Rv0251c |  | Rv2654c |  | Rv1917c |
|  | Rv0570 |  | Rv2030c |  | Rv0331 |  | Rv2656c |  | Rv1978 |
|  | Rv0571c |  | Rv2031c |  | Rv0384c |  | Rv2657c |  | Rv1980c |
|  | Rv0572c |  | Rv2032 |  | Rv0753c |  | Rv2658c |  | Rv2204c |
|  | Rv0573c |  | Rv2488c |  | Rv1130 |  | Rv2659c |  | Rv2244 |
|  | Rv0574c |  | Rv2623 |  | Rv1131 |  | Rv2660c |  | Rv2958c |
|  | Rv0890c |  | Rv2624c |  | Rv1471 | TAS^2^ | Rv0300 |  | Rv2962c |
|  | Rv0894 |  | Rv2625c |  | Rv1472 |  | Rv0301 |  | Rv3347 |
|  | Rv1733c |  | Rv2626c |  | Rv1717 |  | Rv0596 |  | Rv3353c |
|  | Rv1734c |  | Rv2627c |  | Rv1874 |  | Rv1102 |  | Rv3425 |
|  | Rv1735c |  | Rv2628 |  | Rv1875 |  | Rv1103 |  | Rv3429 |
|  | Rv1736c |  | Rv2629 |  | Rv2465c |  | Rv1991c |  | Rv3614c |
|  | Rv1737c |  | Rv2630 |  | Rv2466c |  | Rv2801 |  | Rv3615c |
|  | Rv1738 |  | Rv2631 |  | Rv2662 |  | Rv2901c |  | Rv3849 |
|  | Rv1812c |  | Rv3126c |  | Rv3054c | Others^3,4^ | Rv0009 |  | Rv3865 |
|  | Rv1813c |  | Rv3127 |  | Rv3223c |  | Rv0203 |  | Rv3872 |

|  | | Rv1996 | |  | | Rv3128c | |  | | Rv3307 |  | | | Rv0455c |  | | Rv3873 |
| --- | --- | --- | --- | --- | --- | --- | --- | --- | --- | --- | --- | --- | --- | --- | --- | --- | --- |
|  | Rv1997 | |  | | Rv3129 | |  | | Rv3862c | | |  | Rv0475 | | |  | Rv3878 |
|  | Rv1998c | |  | | Rv3130c | |  | |  | | |  | Rv0642c | | |  | Rv3879c |

NS, Nutrition starvation associated antigens; DosR, Dormancy survival regulon antigens; RA, Reactivation antigens; RPF, Resuscitation-promoting factor; TAS, Toxin-antitoxin system associated antigens.

References:

1. Gordon S V, Eiglmeier K, Garnier T, et al. Genomics of Mycobacterium bovis[J]. Tuberculosis, 2001, 81(1-2):157-163.

2. Ji P, Fan X, Wu K, Lu S. [Research progress on the antigens associated with latent infection of Mycobacterium tuberculosis]. Chinese Journal of Microbiology and Immunology. 2015;35:59-64. (吉萍, 范小勇, 吴康,等. 结核分枝杆菌潜伏性感染相关抗原的研究进展[J]. 中华微生物学和免疫学杂志, 2015(1):59-65.)

3. Chen J, Su X, Zhang Y, Wang S, Shao L, Wu J, et al. Novel recombinant RD2- and RD11-encoded Mycobacterium tuberculosis antigens are potential candidates for diagnosis of tuberculosis infections in BCG-vaccinated individuals. Microbes and infection. 2009;11:876-85.

4. Meier NR, Jacobsen M, Ottenhoff THM, Ritz N. A Systematic Review on Novel Mycobacterium tuberculosis Antigens and Their Discriminatory Potential for the Diagnosis of Latent and Active Tuberculosis. Front Immunol. 2018 Nov 9;9:2476. doi: 10.3389/fimmu.2018.02476. PMID: 30473692; PMCID: PMC6237970.
